# Supplementary material for: Ripple band phase precession of place cell firing during replay
Source: Curr Biol. 2022 Jan 10;32(1):64–73.e5. doi: 10.1016/j.cub.2021.10.033 (PMC8751637; doi:10.1016/j.cub.2021.10.033)
Supplement: Document S1. Figures S1–S5 [file mmc1.pdf]

Current Biology, Volume *32*

Supplemental Information

Ripple band phase precession
# of place cell firing during replay

Daniel Bush, H. Freyja Ólafsdóttir, Caswell Barry, and Neil Burgess

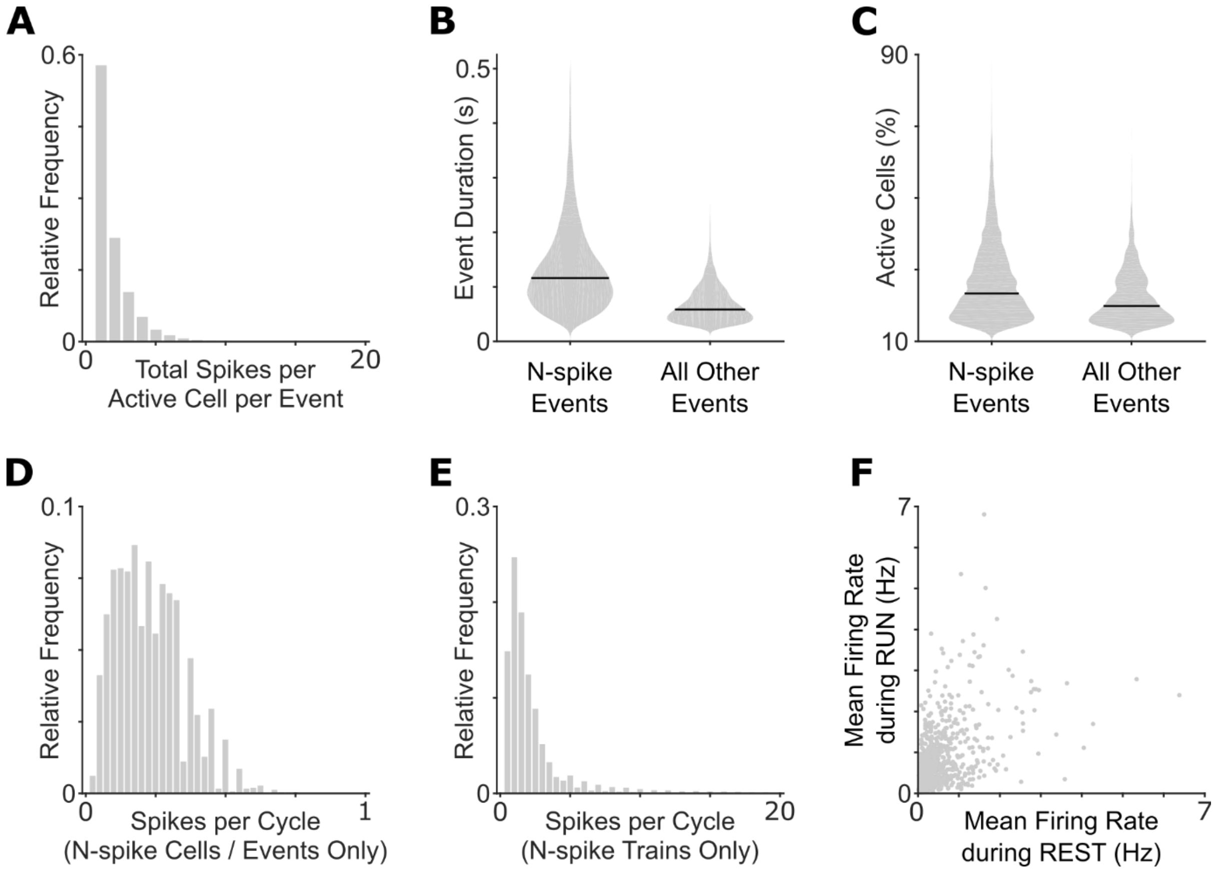

Figure S1: Properties of Candidate Replay Events and Movement Related N-spike Trains, Related to Figure 2

[A] Total number of spikes fired by active place cells within each candidate replay event detected from increased multi-unit activity (MUA) during REST (median±SD = 1±1.4)

[B] Duration of n-spike candidate replay events and all other events. N-spike events tend to be longer (127±72.7 vs. 71±30.9ms; Mann-Whitney U-test, Z = 70, $p<0.001$)

[C] Proportion of active place cells in n-spike candidate replay events and all other events. N-spike events tend to incorporate a greater proportion of active cells (24.1±10.6 vs. 20.7±7.17%; Mann-Whitney U-test, Z = 27.8, $p<0.001$)

[D] Spikes fired per ripple cycle by active n-spike cells in candidate replay events during REST (0.179±0.108)

[E] Spikes fired per theta cycle by active cells in movement related n-spike trains during RUN (1.57±3.0)

[F] Mean firing rates during REST and RUN are highly correlated across place cells ($r = 0.507$, $p<0.001$), although firing rates are significantly higher during RUN (median = 0.458 vs. 0.209Hz, $t(959) = 11.9$, $p<0.001$)

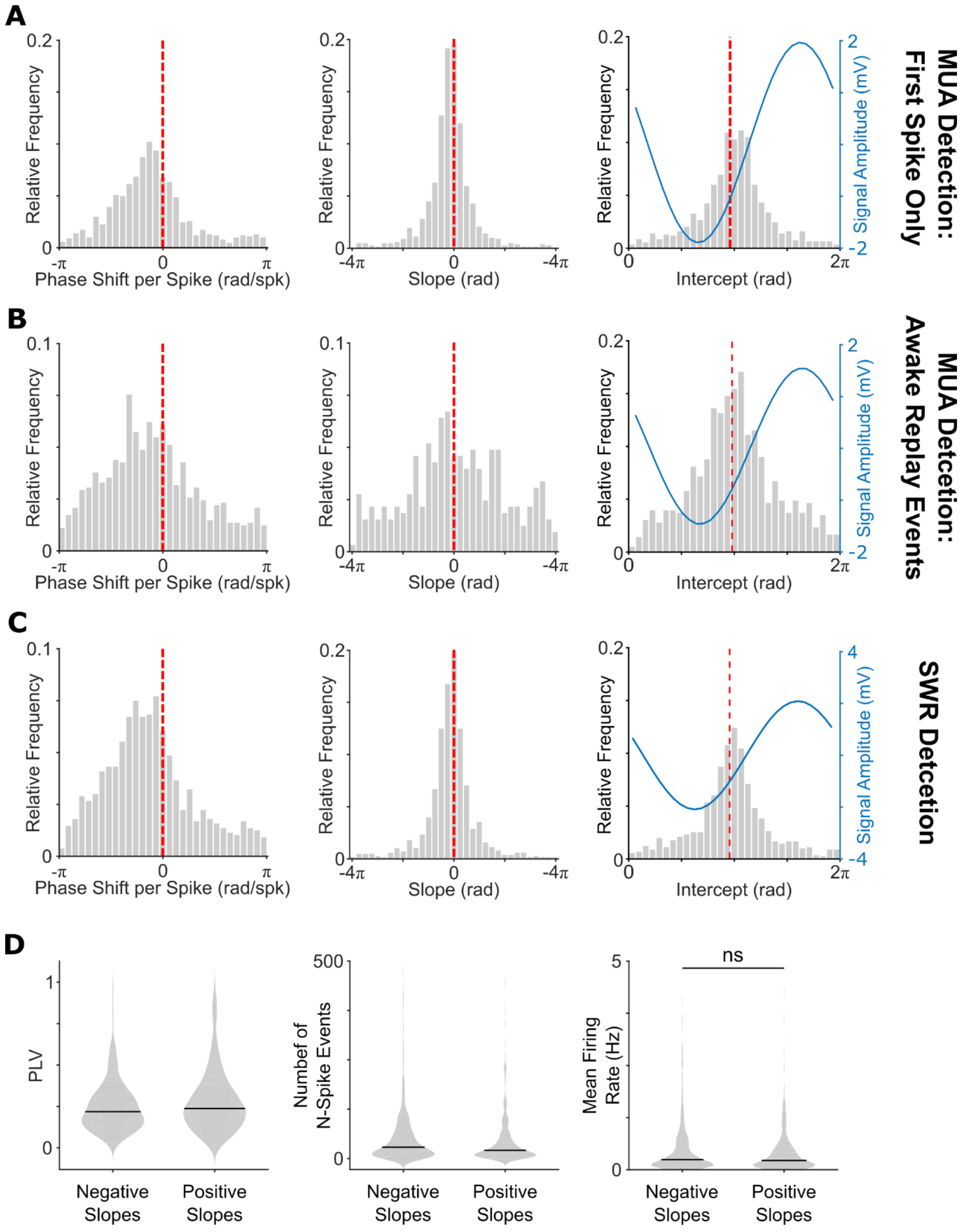
A
B
C
D
MUA Detection: First Spike Only
MUA Detection: Awake Replay Events
SWR Detection
Relative Frequency
Phase Shift per Spike (rad/spk)
Slope (rad)
Intercept (rad)
Signal Amplitude (mV)
PLV
Numbef of N-Spike Events
Mean Firing Rate (Hz)
ns
Negative Slopes
Positive Slopes

Figure S2: Phase Shifts and Within-Event Time vs. Ripple Band Phase Relationships across Behavioural States and Replay Detection Methods, Related to Figure 3

[A] Changes in place cell ripple band firing phase during offline candidate replay events detected from increased MUA, when only the first spike fired in each oscillatory cycle is included. **Left**: Circular mean phase shift between successive spikes across cells (n=951, overall circular median±circular SD=-0.553±1.13 rad/spk). This distribution is non-uniform (Rayleigh test, z=265, p<0.001) with a median value that differs from zero (circular median test, p<0.001); **Middle**: Distribution of within-event time vs. ripple band phase slopes across cells (overall median±SD=-0.459±2.74 rad), which differs from zero (t(947)=-5.91, p<0.001); **Right**: Distribution of intercepts across cells (overall circular mean±circular SD=3.14±1.0 rad). This distribution is non-uniform (Rayleigh test, z=348, p<0.001) with a median value that differs from the preferred firing phase of each cell (3.01±0.79 rad marked with a red dashed line, circular median test, p<0.001)

[B] Changes in place cell ripple band firing phase during online candidate replay events detected from increased MUA. We identified a total of 5590 online replay events using this method (median±SD=133±146 per session, range 62-593) lasting 117±64.4ms with 25±9.91% cells active per event each firing 1±1.56 spikes in total. **Left**: Circular mean phase shift between successive spikes across cells (-0.649±1.51 rad/spk). This distribution is non-uniform (z=84.3, p<0.001) with a median value that differs significantly from zero (p<0.001); **Middle**: Distribution of within-event time vs. ripple band phase slopes across cells (-0.299±3.80 rad), which differs significantly from zero (t(864)=-2.23, p<0.05); **Right**: Distribution of intercepts across cells (3.09±1.31 rad). This distribution is non-uniform (z=155, p<0.001), but the median value does not differ significantly from the preferred firing phase of each cell (3.08±1.08 rad, marked with a red dashed line)

[C] Changes in place cell ripple band firing phase during offline candidate replay events detected from increased ripple band power. We identified a total of 20151 offline replay events using this method (median±SD=686±411 per session, range 112-2175) lasting 155±90.2ms with 25.0±10.6% cells active per event each firing 1±1.31 spikes in total. **Left**: Circular mean phase shift between successive spikes across cells (-0.653±1.33 rad/spk). This distribution is non-uniform (Rayleigh test, z=158.8, p<0.001) with a median value that differs from zero (circular median test, p<0.001); **Middle**: Distribution of within-event time vs. ripple band phase slopes across cells (-0.354±2.91 rad), which differs from zero (t(916)=-4.45, p<0.001); **Right**: Distribution of intercepts across cells (3.03±1.05 rad). This distribution is non-uniform (Rayleigh test, z=303, p<0.001), but the median value does not differ significantly from the preferred firing phase of each cell (3.0±0.783 rad, marked with a red dashed line)

[D] Place **c**ells with negative within-event time v ripple band phase slopes during offline candidate replay events detected from increased MUA were less phase locked to ripple band oscillations (t(948)=2.78, p<0.01) and participated in a greater number of candidate replay events (t(948)=2.13, p<0.05) than cells with positive slopes, without any difference in overall mean firing rates (p=0.21)

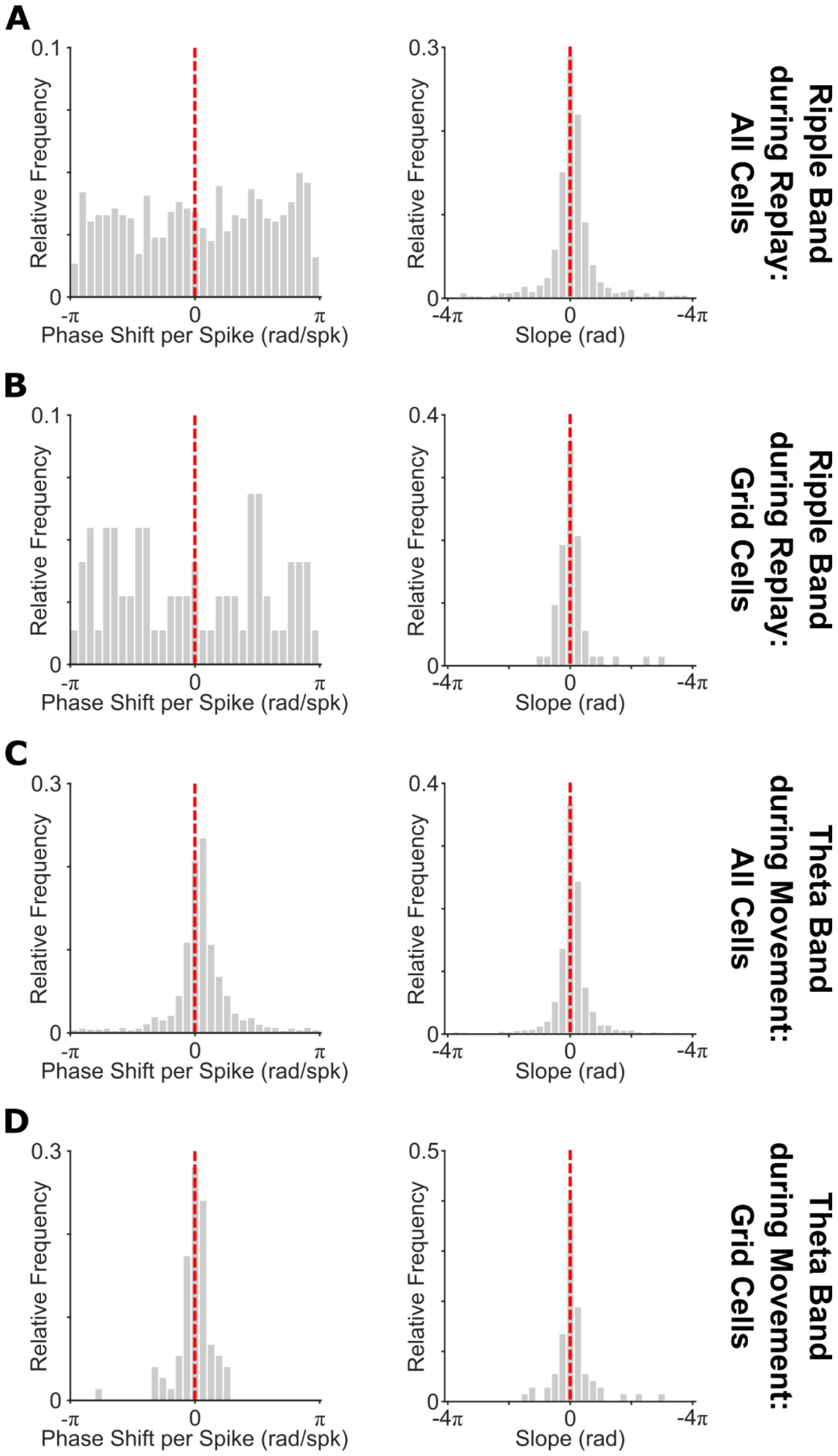
A
B
C
D
Relative Frequency
Phase Shift per Spike (rad/spk)
Slope (rad)
-π
0
π
-4π
0.1
0.3
0.4
0.5
Ripple Band during Replay: All Cells
Ripple Band during Replay: Grid Cells
Theta Band during Movement: All Cells
Theta Band during Movement: Grid Cells

Figure S3: Phase Shifts and Within-Event Time vs. Phase Relationships in MEC, Related to Figures 2 and 3

[A] Changes in the ripple band firing phase of putative principal cells during offline candidate replay events detected from increased MUA in medial entorhinal cortex (MEC). We identified a total of 67540 offline replay events using this method (2701±1144 per session, range 268-4743) lasting 73±54.7ms with 25±9.96% cells active per event each firing 1±1.44 spikes in total. **Left**: Circular mean phase shift between successive spikes across 769/832 n-spike cells. This distribution is non-uniform (Rayleigh test, $z=3.02$, $p=0.05$) with a median value that does not differ from zero (circular median test, $p=0.15$), but does differ from the phase shift across place cells (Watson-Williams test, $F=215$, $p<0.001$); **Right**: Distribution of within-event time vs. ripple band phase slopes across cells (0.172±2.52 rad), which differs both from zero ($t(768)=2.63$, $p<0.01$) and from slopes across place cells ($t(1737)=-6.44$, $p<0.001$)

[B] Changes in the ripple band firing phase of grid cells during offline candidate replay events detected from increased MUA in MEC. **Left**: Circular mean phase shift between successive spikes across 73/832 n-spike grid cells. This distribution is uniform (Rayleigh test, $z=0.848$, $p=0.43$) and differs from the phase shift across place cells (Watson-Williams test, $F=26.5$, $p<0.001$); **Right**: Distribution of within-event time vs. ripple band phase slopes across cells (-0.119±1.83 rad), which is not different from zero ($t(72)=1.13$, $p=0.26$) but does differ from slopes across place cells ($t(1042)=-2.66$, $p<0.01$)

[C] Changes in the theta firing phase of putative principal cells during movement related n-spike trains in MEC. We identified a total of 121538 spike trains (4635±1936 per session, range 856-7218) lasting 1.53±1.37s and incorporating 5.0±6.66 spikes per train. **Left**: Circular mean phase shift between successive spikes across 1011/1033 n-spike cells (0.171±0.714 rad/spk). This distribution is non-uniform (Rayleigh test, $z=607$, $p<0.001$) with a median value that differs from zero (circular median test, $p<0.001$); **Right**: Distribution of within-event vs. theta phase slopes across cells (0.185±1.74 rad), which differs from zero ($t(1008)=3.99$, $p<0.001$)

[D] Changes in the theta firing phase of grid cells during movement related n-spike trains in MEC. **Left**: Circular mean phase shift between successive spikes across 75/1033 n-spike grid cells (0.0133±0.457 rad/spk). This distribution is non-uniform (Rayleigh test, $z=60.8$, $p<0.001$) with a median value that does not differ from zero (circular median test, $p=0.49$); **Right**: Distribution of within-event time vs. theta phase slopes across cells (0.119±2.02 rad), which is not different from zero ($t(74)=1.08$, $p=0.29$)

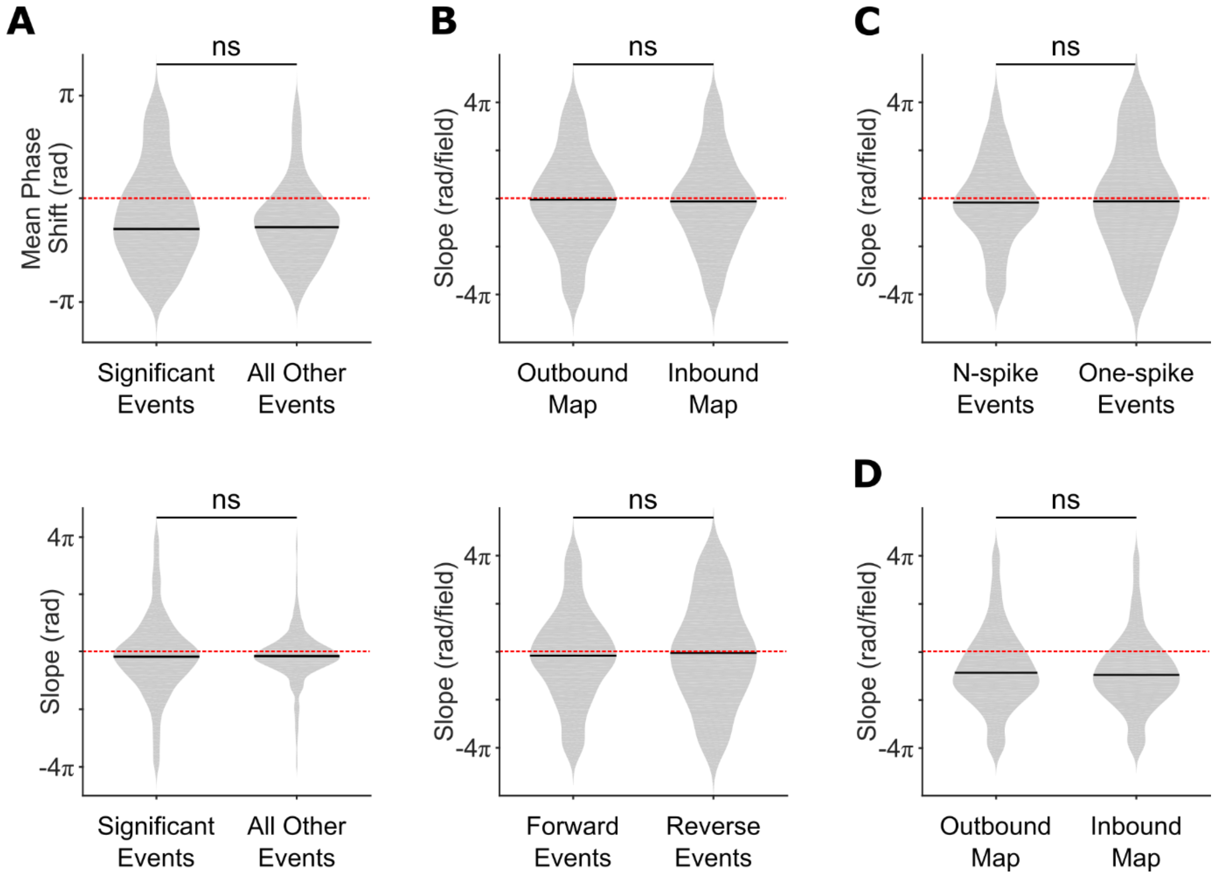

Figure S4: Comparing Phase Precession across Replay Events, Trajectory Directions and Firing Rate Maps, Related to Figures 3 and 4

[A] Comparison of within-event phase shift and time vs. ripple band phase slopes between significant and non-significant linear replay events. There was no difference in either phase shifts per spike pair (**upper panel:** circular median test, p=0.54; significant=-0.936±1.50, non-significant=-0.882±1.13 rad/spk) or slopes (**lower panel:** t(779)=-0.048, p=0.96; significant-0.559±4.30, non-significant=-0.506±2.37 rad) between these events across cells

[B] Comparison of within-field location vs. ripple band phase slopes between significant events on the outbound and inbound map; forward and reverse events. There was no difference in slopes between the outbound and inbound maps, for cells with fields included on both maps (**upper panel:** t(277)=-0.596, p=0.55; outbound=-0.201±6.09, inbound=-0.437±6.03 rad/field); or by forward or reverse trajectories, for cells with sufficient spikes fired in each decoded movement direction through the same field (**lower panel:** t(343)=-0.825, p=0.41; forward=-0.556±6.03, reverse=-0.201±6.07 rad/field)

[C] Comparison of within-field location vs. ripple band phase slopes between n-spike and one-spike events. There was no difference in slopes computed using events in which each place cell fired ≥3 spikes and events in which each place cell fired just one spike, across cells with sufficient in-field spikes in both cases (t(150)=0.158, p=0.88; n-spike events=-0.614±5.77, one-spike events=-0.478±6.90 rad/field)

[D] Comparison of within-field location vs. theta phase slopes between the outbound and inbound map. There was no difference in slopes between the outbound and inbound maps, for cells with fields included on both maps (t(537)=1.43, p=0.15; outbound=-2.78±5.24, inbound=-3.07±4.89 rad/field)

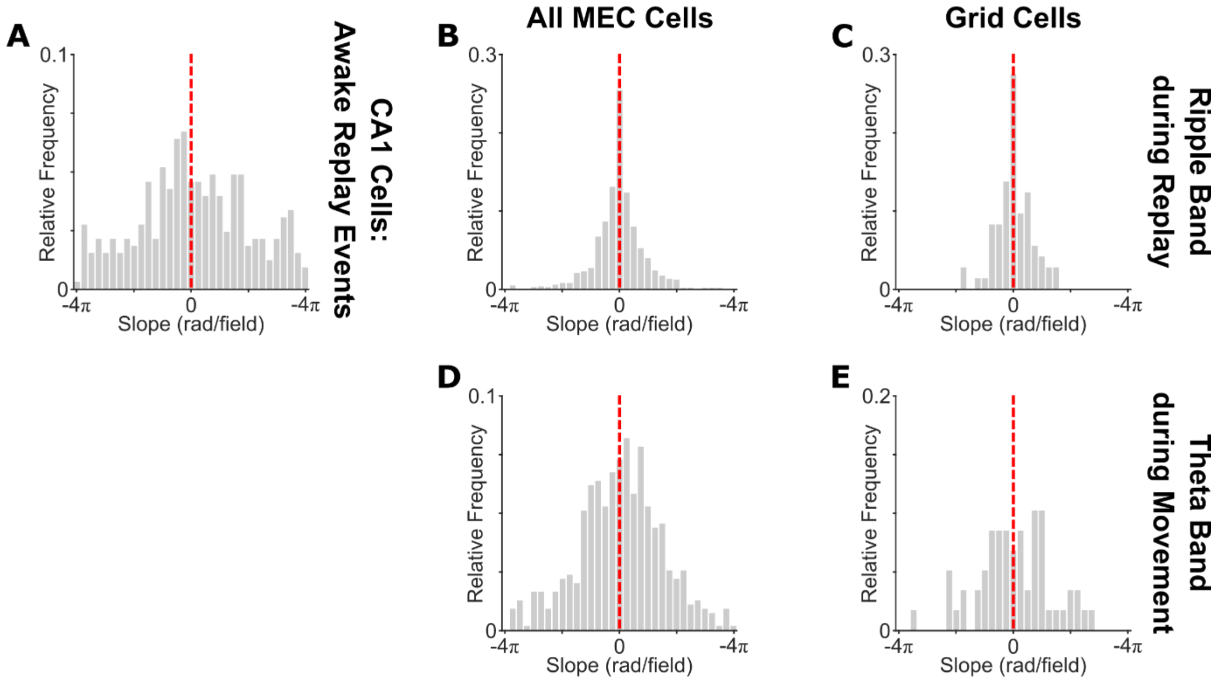

Figure S5: Within-Field Location vs. Firing Phase Relationships during Online Replay Events and in MEC, Related to Figure 4

[A] Within-field location vs. ripple band firing phase slopes across CA1 place cells during online candidate replay events detected from increased MUA. A total of 1177/5590 online replay events corresponded to significant linear trajectories on the track (20±9.16% events per session); and a total of 721 place fields subsequently passed our threshold for inclusion in this analysis (≥5 spikes covering ≥50% of the firing field), with 360/1044 cells (34.5%) having ≥1 field included and 12±12.3 spikes per field (range 5-115). The distribution of slopes across cells (-0.289±6.97 rad/field) does not differ from zero (t(359)=0.067, p=0.95)

[B] Within-field location vs. ripple band firing phase slopes across putative principal cells in MEC during offline candidate replay events detected from increased MUA. A total of 7211/67540 offline replay events corresponded to significant linear trajectories on the track (10.9±3.11% events per session); and a total of 2442 firing fields subsequently passed our threshold for inclusion in this analysis (≥5 spikes covering ≥50% of the firing field), with 572/877 cells (65.2%) having ≥1 field included and 16±37.4 spikes per field (range 5-602). The distribution of slopes across cells (0.299±4.65 rad/field) does not differ from zero (t(550)=0.326, p=0.744)

[C] Within-field location vs. ripple band firing phase slopes across MEC grid cells during offline candidate replay events detected from increased MUA. A total of 59/877 grid cells passed our criteria for inclusion in this analysis. The distribution of slopes across these cells (-0.00628±4.02 rad/field) does not differ from zero (t(58)=0.310, p=0.758)

[D] Within-field location vs. theta firing phase slopes across putative principal cells in MEC during RUN. A total of 945/1033 cells A total of 5498 firing fields passed our threshold for inclusion in this analysis (≥5 spikes covering ≥50% of the firing field), with 946/1033 cells (91.6%) having ≥1 field included and 65±128 spikes per field (range 5-1251). The distribution of slopes across cells (-0.00628±2.56 rad/field) does not differ from zero (t(944)=-0.753, p=0.452)

[E] Within-field location vs. theta firing phase slopes across MEC grid cells during RUN. A total of 73/1033 grid cells passed our criteria for inclusion in this analysis. The distribution of slopes across these cells (0.0792±2.03 rad/field) does not differ from zero (t(72)=0.344, p=0.732). However, 78/536 grid fields (14.6%) showed a significant negative correlation between within-field location and theta phase, which is more than expected by chance (binomial test, p<0.001)
