# Supplementary material for: Ripple band phase precession of place cell firing during replay
Source: Curr Biol. 2022 Jan 10;32(1):64–73.e5. doi: 10.1016/j.cub.2021.10.033 (PMC8751637; doi:10.1016/j.cub.2021.10.033)
Supplement: Document S2. Article plus supplemental information [file mmc2.pdf]

Current Biology

Article

Ripple band phase precession of place cell firing during replay

Graphical abstract

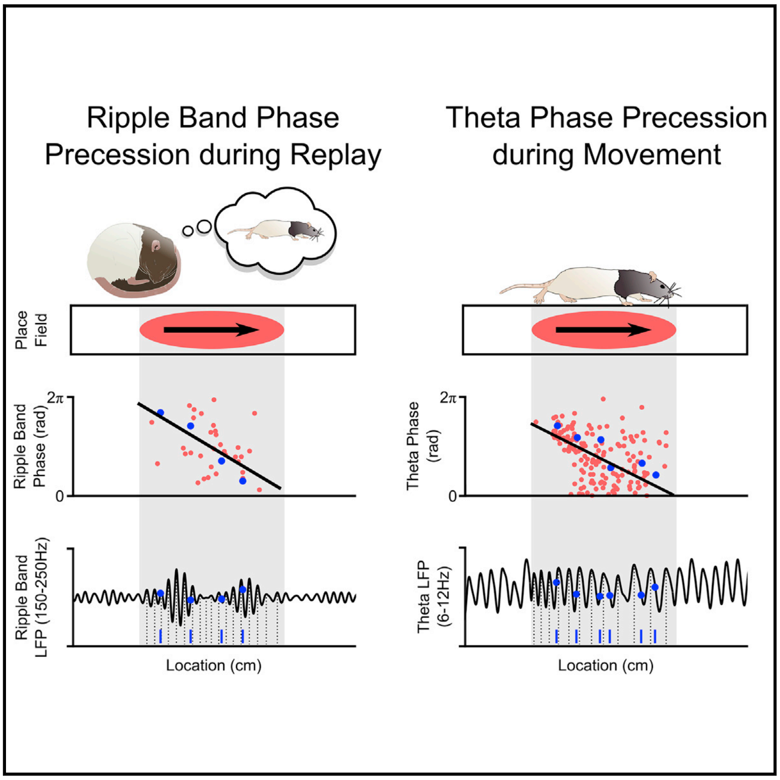

Authors

Daniel Bush, H. Freyja Ólafsdóttir, Caswell Barry, Neil Burgess

Correspondence

drdanielbush@gmail.com (D.B.),
caswell.barry@ucl.ac.uk (C.B.)

In brief

Bush et al. report that, during hippocampal replay events, excitatory CA1 neurons display phase precession relative to the ripple oscillation, similar to the well-characterized theta phase precession observed during active movement. At the network level, this produces forward sweeps of activity during each ripple cycle that encode movement direction.

Highlights

- Place cells fire at successively earlier ripple band phases during replay
- Ripple band firing phase during replay encodes location within the place field
- This produces forward sweeps of place cell activity during each ripple cycle

Bush et al., 2022, Current Biology *32*, 64–73
January 10, 2022 © 2021 The Author(s). Published by Elsevier Inc.
https://doi.org/10.1016/j.cub.2021.10.033

CellPress

CellPress
OPEN ACCESS

Current Biology

Article

Ripple band phase precession of place cell firing during replay

Daniel Bush,[1,2,5,7,*] H. Freyja Ólafsdóttir,[3,5] Caswell Barry,[4,6,*] and Neil Burgess[1,2,6]
[1]UCL Institute of Cognitive Neuroscience, Queen Square, London, UK
[2]UCL Institute of Neurology, Queen Square, London, UK
[3]Donders Institute for Brain Cognition and Behaviour, Radboud University, Nijmegen, the Netherlands
[4]UCL Department of Cell and Developmental Biology, Gower Street, London, UK
[5]These authors contributed equally
[6]These authors contributed equally
[7]Lead contact
*Correspondence: drdanielbush@gmail.com (D.B.), caswell.barry@ucl.ac.uk (C.B.)
https://doi.org/10.1016/j.cub.2021.10.033

SUMMARY

Neuronal "replay," in which place cell firing during rest recapitulates recently experienced trajectories, is thought to mediate the transmission of information from hippocampus to neocortex, but the mechanism for this transmission is unknown. Here, we show that replay uses a phase code to represent spatial trajectories by the phase of firing relative to the 150- to 250-Hz "ripple" oscillations that accompany replay events. This phase code is analogous to the theta phase precession of place cell firing during navigation, in which place cells fire at progressively earlier phases of the 6- to 12-Hz theta oscillation as their place field is traversed, providing information about self-location that is additional to the rate code and a necessary precursor of replay. Thus, during replay, each ripple cycle contains a "forward sweep" of decoded locations along the recapitulated trajectory. Our results indicate a novel encoding of trajectory information during replay and implicates phase coding as a general mechanism by which the hippocampus transmits experienced and replayed sequential information to downstream targets.

INTRODUCTION

The mammalian hippocampus is implicated in both spatial cognition and episodic memory function.[1,2] In the rodent hippocampal formation, place and grid cells are active in restricted regions of space—the corresponding place or grid field.[3,4] During active movement, when 6- to 12-Hz theta oscillations dominate the local field potential (LFP), place cells and a subset of grid cells in medial entorhinal cortex (MEC) also exhibit a theta phase code for location. Specifically, these cells fire at progressively earlier phases of the theta cycle as the firing field is traversed.[5,6] Because phase precession is coordinated across cells, this produces theta "sweeps" of activity at the network level that encode a sequence of locations beginning behind and progressing ahead of the animal within each oscillatory cycle.[7–9] The theta phase code for location exhibited by place and grid cells improves the accuracy of decoding location[10] and allows movement direction to be inferred from population activity in each oscillatory cycle.[11,12]

Importantly, however, phase is independent of frequency, and a similar coding scheme could therefore be supported by oscillatory activity in other frequency bands, such as 25- to 55-Hz slow gamma,[13–15] or by an LFP signal whose instantaneous frequency varies dynamically over a wide range, such as that observed in bats and humans.[12,16–18] During periods of quiescent waking and rest, the hippocampal LFP exhibits prominent sharp-wave ripple (SWR) events composed of a large-amplitude deflection accompanied by a transient increase in 150- to 250-Hz ripple band power.[19] SWR events are associated with prominent place cell multi-unit activity (MUA), which can recapitulate coherent spatial trajectories through recently visited environments.[20–22] Here, we asked whether place cells might also exhibit phase coding relative to ripple band oscillations during replay events. Specifically, this should be characterized by a systematic change in ripple band firing phase across multiple spikes fired by individual place cells within each candidate replay event, a relationship between decoded location within the firing field and ripple band firing phase across multiple replay events through the same place field, and a relationship between ripple band phase and the relative location encoded by population activity across multiple place cells within each replay event.

RESULTS

We analyzed data from rats completing shuttle runs along a linear track for food reward and during subsequent sleep (as described previously).[23,24] Six animals undertook a total of 29 RUN sessions lasting 34.4 ± 11.7 min (median ± SD; range 21.6–60.9) on different days. During RUN sessions, animals successfully completed 20 ± 5.7 outbound and inbound runs along a 6-m Z-shaped track (Figure 1A) while we recorded the activity of 34 ± 17.7 putative pyramidal cells in dorsal CA1 (14–71 per session, 1,044 in total). After each RUN session, animals rested for

 © 2021 The Author(s). Published by Elsevier Inc.
This is an open access article under the CC BY license (http://creativecommons.org/licenses/by/4.0/).

CellPress
OPEN ACCESS

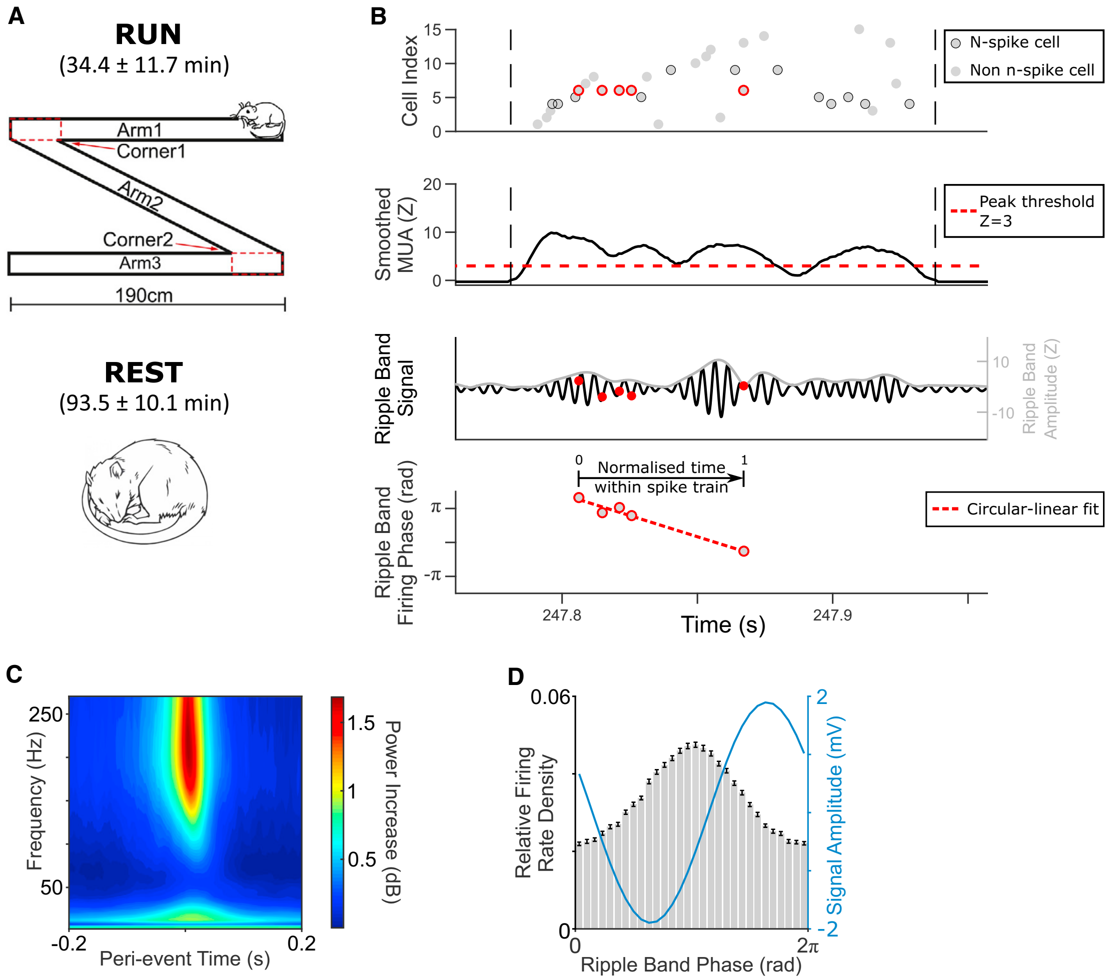

Figure 1. Candidate replay events during REST

(A) Task schematic.

(B) Schematic analysis of an "n-spike" event; spikes highlighted in red correspond to the multiple spikes fired by a single n-spike cell and plotted in the bottom panels.

(C) Average wavelet spectrogram across all candidate replay events in all sessions, time locked to peak multi-unit activity (MUA) within each event.

(D) Relative firing rate (gray bars) and mean LFP signal amplitude by ripple band phase (blue line), averaged across all candidate replay events in all sessions.

93.5 ± 10.1 (90–129) min (REST) while we continued to record the activity of 30 ± 17.8 putative pyramidal cells in dorsal CA1 (14–76 per session, 1,025 in total, including 960 that were also active during RUN) alongside LFP at a high sample rate (4.8 kHz). Putative interneurons, identified by narrow waveforms and mean firing rates >10 Hz, were excluded from all analyses.

First, we looked for candidate replay events during REST on the basis of MUA (see STAR Methods for further details).[24] This identified a total of 25,328 events (758 ± 599 per session, range 97–3,113, equivalent to events occurring at a rate of ~0.13 Hz). Candidate events lasted 114 ± 71.3 ms and incorporated activity in 23.3% ± 10.2% of all recorded pyramidal cells (range 15.0%–86.2%; see Figure 1B for an example). During candidate replay events, increased MUA was accompanied by elevated power in the 150- to 250-Hz ripple band (Figure 1C), and the firing rate of 69.4% of pyramidal cells was significantly modulated by ripple band phase (Figure 1D). Although active cells typically fired only a single spike each during candidate replay events (median ± SD = 1 ± 1.4; Figure S1A), multiple cells were active during each event. Hence, to examine changes in the ripple band firing phase of individual cells over the course of each event, we identified a subset of "n-spike" events during which one or more active cells fired ≥3 spikes each. The vast majority of recorded cells (93.0%) participated in at least one n-spike event, the majority of candidate replay events (20,224 or 79.9%) were n-spike events (range 60.3%–94.0% per session), and n-spike events tended to be longer and incorporate more active cells than other events (Figures S1B and S1C).

Next, we estimated the ripple band firing phase of each spike by applying the Hilbert transform to LFP data filtered in the 150- to 250-Hz range—shifting the resultant phase values so that π rad corresponded to the circular mean firing phase of all cells recorded in each session. We then computed the ripple band phase shift between successive pairs of spikes fired by n-spike

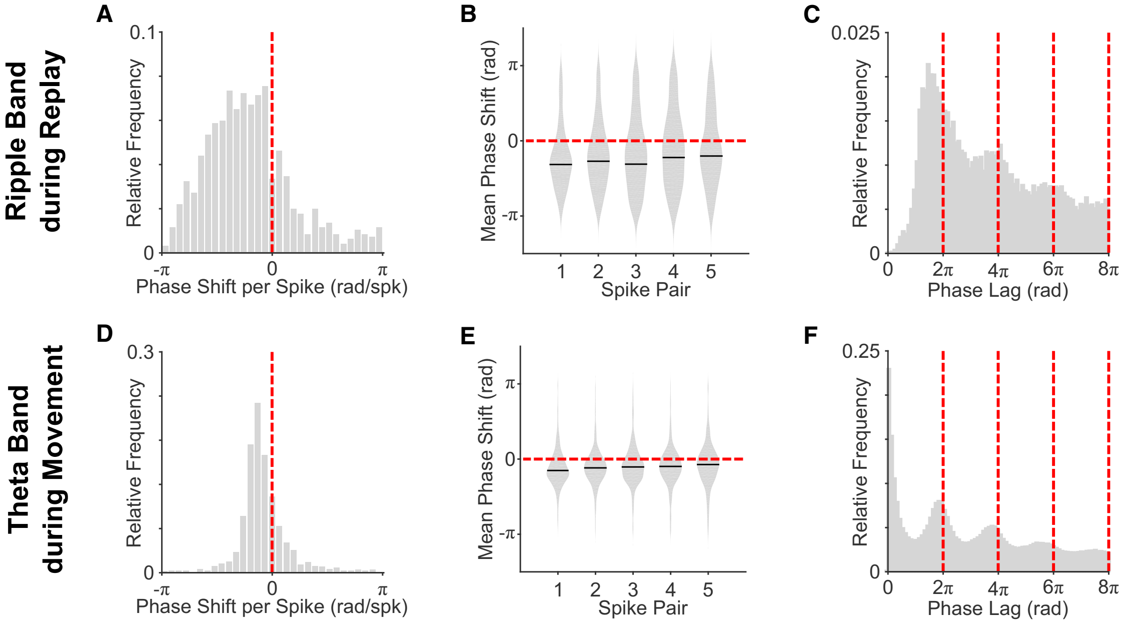

Figure 2. Ripple band firing phase shift during candidate replay events in REST and theta firing phase shift during movement-related n-spike trains in RUN

(A) Circular mean ripple band phase shift between all successive spikes in candidate replay events across n-spike cells (n = 953; overall circular median ± circular SD = −0.922 ± 1.19 rad/spk). This distribution is non-uniform (Rayleigh test; $Z = 229$; $p < 0.001$) with a median value that differs from zero (circular median test; $p < 0.001$).

(B) Circular mean ripple band phase shift by within-event spike pair, averaged across n-spike cells. Each phase shift is non-uniformly distributed with a median value that differs from zero (all $p < 0.001$).

(C) Ripple band firing phase auto-correlogram, averaged across n-spike cells.

(D) Circular mean theta phase shift between successive spikes in movement-related n-spike trains (−0.307 ± 0.61 rad/spk). This distribution is non-uniform (Rayleigh test; $Z = 673$; $p < 0.001$) with a median value that differs from zero (circular median test; $p < 0.001$).

(E) Circular mean theta phase shift by within-event spike pair, averaged across all cells. Each phase shift is non-uniformly distributed with a median value that differs from zero (all $p < 0.001$).

(F) Theta firing phase auto-correlogram, averaged across cells.

See also Figures S1 and S3.

cells and averaged those phase shifts across all spike pairs and then across all n-spike events. Remarkably, we found that these phase shifts were consistently negative (Figure 2A). Specifically, the distribution of circular mean phase shifts across cells was non-uniform, with an overall circular median that differed significantly from zero. Importantly, this was true when each spike pair (i.e., first, second, third, etc.) was considered separately, indicating that the overall effect was consistent across the spike train (Figure 2B). This was supported by an inspection of the ripple band firing phase auto-correlogram during n-spike events,[17,25] which shows multiple peaks with phase lags faster than the LFP (Figure 2C).

In addition, we used circular-linear regression to estimate the slope and intercept of the relationship between normalized time within the spike train (i.e., where the time of the first and last spike was 0 and 1, respectively) and ripple band phase for each n-spike cell in each n-spike event.[26] Consistent with the results above, the median slope of this within-event time versus ripple band phase relationship was negative and significantly different from zero across cells (Figure 3A). The distribution of intercepts was also non-uniform, with a circular mean value that was slightly but significantly later in the ripple cycle than the mean firing phase of all n-spike cells (Figure 3B), consistent with a shift to earlier phases when spikes occur later in the train. Hence, the ripple band firing phase of putative place cells during candidate replay events begins just after the phase of peak firing and becomes progressively earlier as the event continues (Figure 3C).

Importantly, when analyzed separately, each animal exhibited negative circular mean phase shifts (range of −1.57 to −0.43 rad/spk; circular median test; $p < 0.05$) and mean slopes (range of −0.657 to −0.394 rad; $t(5) = -15.1$; $p < 0.001$) averaged across all n-spike cells. Moreover, because n-spike cells typically fire <1 spike per ripple band cycle (Figure S1D), results were similar if we included only the first spike from each oscillatory cycle in these analyses (Figure S2A). Qualitatively similar results were also obtained when analyzing candidate “online” replay events, which occurred while the animal was awake but immobile at the corners of the track during RUN, indicating that this phenomenon is preserved across behavioral states (Figure S2B), and when candidate replay events were identified using ripple band power instead of MUA (Figure S2C; see STAR Methods for further details). Conversely, we observed no consistent change in ripple band firing phase across n-spike trains fired by putative principal neurons recorded simultaneously in the deeper layers of medial entorhinal cortex (MEC) or if we restricted that analysis to just grid cells (Figures S3A and S3B).

CellPress
OPEN ACCESS

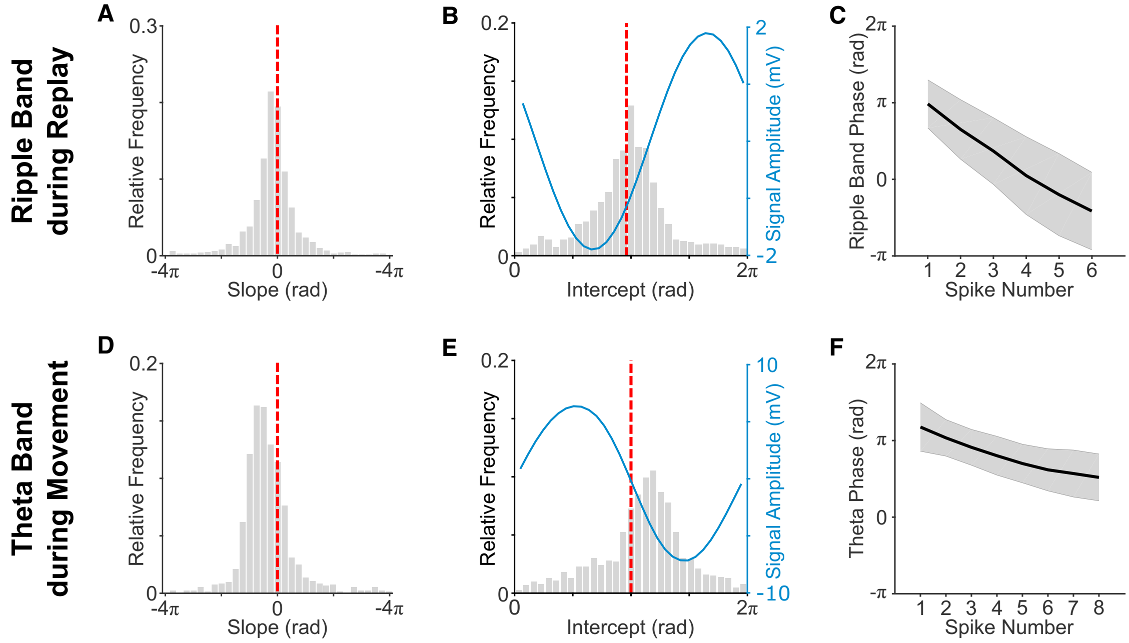

Figure 3. Within-event time versus ripple band firing phase during candidate replay events in REST and within-event time versus theta phase during movement-related n-spike trains in RUN

(A) Distribution of within-event time versus ripple band phase slopes across cells (see Figure 1B for an example; overall median ± SD = −0.502 ± 2.66 rad), which differs from zero ($t(949) = -6.27$; $p < 0.001$).

(B) Distribution of within-event time versus ripple band phase intercepts across cells (see Figure 1B for an example; overall circular mean ± circular SD = 3.06 ± 1.04 rad), alongside mean LFP signal amplitude by ripple band phase (blue line). This distribution is non-uniform (Rayleigh test; $Z = 323$; $p < 0.001$) with a median value that differs from the preferred firing phase of each cell (3.01 ± 0.79 rad, indicated by the dashed red line; circular median test; $p < 0.01$).

(C) Unwrapped ripple band phase by spike number during candidate replay events, averaged across cells (error bars indicate circular SD).

(D) Distribution of within-event time versus theta phase slopes (−1.44 ± 2.77 rad), which differs from zero ($t(977) = -13.1$; $p < 0.001$).

(E) Distribution of within-event time versus theta phase intercepts across cells (3.59 ± 1.12 rad), alongside mean LFP signal amplitude by theta phase (blue line). This distribution is non-uniform (Rayleigh test; $Z = 285$; $p < 0.001$) with a median value that differs from the preferred firing phase of each cell (3.14 ± 1.17 rad, indicated by the dashed red line; circular median test; $p < 0.001$).

(F) Unwrapped theta phase by spike number within movement-related n-spike trains, averaged across cells (error bars indicate circular SD).

See also Figures S2–S4.

To further characterize this phenomenon, we carried out several control analyses. First, we examined whether these results might be accounted for by bursting, which is common in CA1 pyramidal cells and can produce inter-spike intervals that are slightly shorter than a typical ripple cycle (~5 ms). However, we found no evidence for any relationship between the bursting index and average ripple band phase shifts or within-event time versus ripple band firing phase slopes across cells (both $p > 0.11$). Second, we examined whether these results differed between cells that were recorded on the same tetrode as the LFP signal ($n = 59$) and those recorded on a different tetrode ($n = 966$) but found no difference in preferred firing phases, average phase shifts, slopes, or intercepts of the within-event time versus ripple band firing phase relationship between groups (all $p > 0.1$). Similarly, we asked whether these results were affected by using a different, local LFP signal, where one was available ($n = 63$ cells), but found no change in ripple band phase shifts, slopes, or intercepts of the within-event time versus ripple band firing phase relationship (all $p > 0.16$). Finally, although most cells (605/1,025) exhibited a negative within-event time versus ripple band phase slope, a substantial number (345/1,025) exhibited a positive slope, and we therefore sought to identify any differences between these two populations. We found that cells with negative slopes were less phase locked to ripple band oscillations and participated in a greater number of candidate replay events, without any difference in overall mean firing rates (Figure S2D).

Next, for comparison, we used analogous methods to examine changes in theta firing phase across spike trains from the same cells during active movement on the track. Specifically, we looked for extended periods of elevated activity in each cell during movement, independent of spatial location, and inspected the relationship between theta firing phase—again, defined such that π rad corresponds to the circular mean preferred firing phase of all cells recorded in each session—and spike number or normalized time within each n-spike train (see STAR Methods for further details).[27] During these movement-related n-spike trains, the firing rate of 37.1% of pyramidal cells was significantly modulated by theta phase. In this case, because active cells typically fire >1 spike per theta cycle (Figure S1E), we restricted our subsequent analyses to the first spike from each oscillatory cycle.

In accordance with previous studies, and similar to the changes in ripple band firing phase during candidate replay

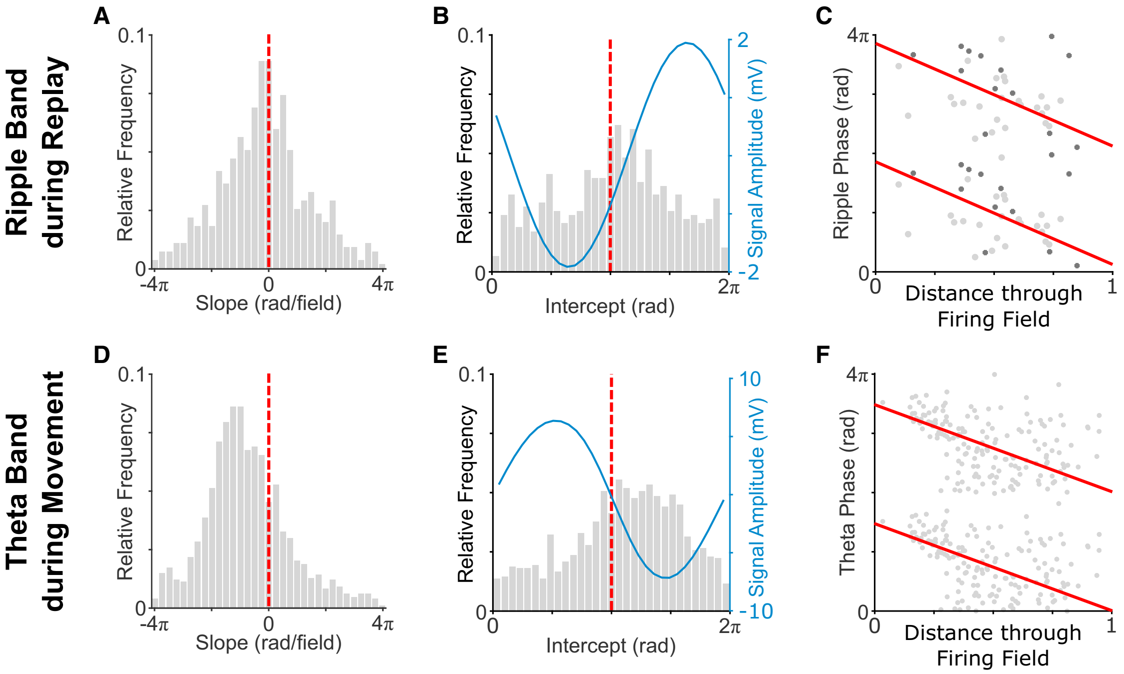

Figure 4. Within-field location versus ripple band firing phase during replay events in REST and within-field location versus theta phase during movement in RUN

(A) Distribution of within-field location versus ripple band phase slopes (overall median ± SD = −0.505 ± 4.80 rad/field), which differs from zero (t(553) = −2.36; p = 0.0184).

(B) Distribution of within-field location versus ripple band phase intercepts (overall circular median ± circular SD = 3.42 ± 1.78 rad), alongside mean LFP signal amplitude by ripple band phase (blue line). This distribution is non-uniform (Rayleigh test; $Z = 24.0$; $p < 0.001$) with a median value that differs from the phase of peak firing (3.12 ± 0.61, indicated with a dashed red line; circular median test; $p < 0.001$).

(C) Decoded within-field location versus ripple band firing phase relationship for a typical place field, incorporating spikes from both n-spike events (light gray) and events where the cell fired only a single spike (dark gray), alongside the circular-linear fit (red line).

(D) Distribution of within-field location versus theta phase slopes (−2.77 ± 3.94 rad/field), which differs from zero (t(856) = −18.0; $p < 0.001$).

(E) Distribution of within-field location versus theta phase intercepts (3.90 ± 1.63 rad), alongside mean LFP signal amplitude by theta phase (blue line). This distribution is non-uniform (Rayleigh test; $Z = 99.6$; $p < 0.001$) with a median value that differs from the phase of peak firing (3.20 ± 1.17 rad, indicated by a dashed red line; circular median test; $p < 0.001$).

(F) Within-field location versus theta firing phase relationship for the place field shown in (C), alongside the circular-linear fit (red line).

See also Figures S4 and S5.

events described above, theta phase shifts between successive spikes were consistently negative (Figure 2D). Specifically, the distribution of circular mean phase shifts across putative place cells was non-uniform, with an overall circular mean that differed significantly from zero. Importantly, this effect was true when each spike pair (i.e., first, second, third, etc.) was considered separately (Figure 2E) and is supported by an inspection of the average theta firing phase auto-correlogram, which exhibits multiple peaks with phase lags faster than the LFP (Figure 2F). The relationship between normalized time within movement-related n-spike trains and theta phase also had a consistently negative slope that was significantly different from zero across cells (Figure 3D), and the distribution of intercepts was non-uniform, peaking after the phase of maximum firing in the theta cycle (Figure 3E). In sum, these results indicate that the theta phase of putative place cell firing during movement begins after the phase of peak firing in the theta cycle and becomes progressively earlier as the spike train continues (Figure 3F). Conversely, consistent changes in theta band firing phase were not observed across putative principal neurons recorded simultaneously in the deeper layers of MEC or if we restricted that analysis to just grid cells (Figures S3C and S3D).

Next, to establish whether putative place cells exhibit similar firing phase shifts with respect to different LFP oscillations during different behavioral states, we directly compared within-event time versus phase relationships between candidate replay events during REST and movement-related n-spike trains during RUN across cells. Interestingly, across cells that participated in ≥1 n-spike event in both REST and RUN (n = 853), within-event time versus phase slopes were correlated between sessions ($r = 0.0713$; $p < 0.05$), indicating that cells that exhibited larger changes in ripple band firing phase across n-spike trains during individual replay events tended to exhibit larger changes in theta firing phase across individual movement-related n-spike trains during active movement. In addition, mean firing rates were highly correlated between REST and RUN, although firing rates were significantly higher during RUN (Figure S1E). Importantly, however, we found no evidence for a sub-population of place cells that consistently display positive within-event time versus firing phase slopes relative to both

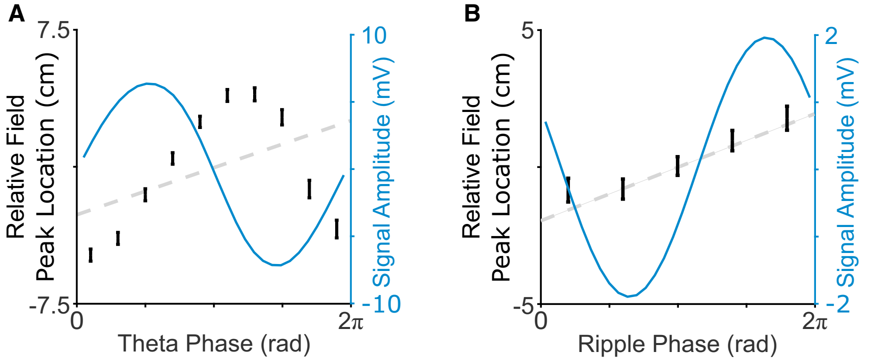

Figure 5. Forward sweeps of place cell activity in theta cycles during RUN and ripple band cycles during REST

(A) Overall theta sweep, showing median relative distance to the peak of the nearest firing field (black error bars) across all active place cells alongside actual location (dashed gray line) and mean LFP signal amplitude (blue line) in each of 10 theta phase bins. On average, forward sweeps cover 13.2 ± 51.4 cm within each theta cycle, which is significantly different from zero across events ($t(5,441) = 17.3$; $p < 0.001$), corresponding to a movement speed of 108 ± 504 cm/s, which significantly exceeds the animal's actual movement speed of 43.6 ± 16.7 cm/s during the same events ($t(5,441) = 10.9$; $p < 0.001$).

(B) Overall ripple sweep, showing median relative distance to the peak of the nearest firing field across all active place cells alongside decoded location (dashed gray line) and mean LFP signal amplitude (blue line) in each of 5 ripple band phase bins. On average, forward sweeps cover 2.61 ± 56.3 cm within each ripple cycle, which is significantly different from zero across events ($t(3,404) = 4.29$; $p < 0.001$), corresponding to a movement speed of 6.02 ± 134 m/s, which is not significantly different from the decoded trajectory speed during the same events ($t(3,404) = -0.53$; $p = 0.59$).

ripple oscillations during REST and theta oscillations during RUN ($\chi^2 = 1.93$; $p = 0.17$).

Crucially, previous studies have demonstrated that place cell theta firing phase during movement does not simply change as a function of spike number or time since firing began but is most strongly modulated by distance traveled through the place field.[5] As a result, theta firing phase encodes information about the animal's location, beyond that provided by firing rates alone.[10,28] In addition, theta firing phase encodes information about movement direction, even when this conflicts with the head direction signal.[12,29–31] Next, we sought to examine whether ripple band firing phase shared a similar relationship with decoded location within the place field during replay events.

To establish this, we first defined place fields as ≥10 contiguous 2-cm spatial bins with smoothed firing rate greater than the mean across all bins and a peak firing rate of ≥1 Hz. In total, 919/1,044 putative pyramidal cells (88%) active during RUN having ≥1 field on either outbound or inbound runs along the track. We then sought to identify which candidate replay events encoded coherent spatial trajectories on the Z-maze using a Bayesian decoding algorithm and trajectory fitting procedure (see STAR Methods for further details).[32] This revealed that 13.9% of all candidate events (3,523/25,328; 13.5% ± 4.7% across sessions; range 4.21%–24.9%) corresponded to the replay of linear trajectories along the track. Interestingly, there was no difference in ripple band phase shifts per spike pair or within-event time versus ripple band firing phase slopes between significant and non-significant linear replay events across cells (Figure S4A). Finally, we used these fitted trajectories to estimate the mean decoded location in each ripple cycle during significant linear replay events. For each place field, we could then examine the relationship between ripple band firing phase and decoded location within the firing field in the corresponding ripple band cycle, collapsed across all significant decoded trajectories that passed through that firing field in the same direction of movement (i.e., considering forward and reverse replay events separately).

We identified a total of 1,569 place fields that passed our criteria for inclusion (≥5 spikes fired within the field across all significant decoded trajectories through that field, covering ≥50% of the place field), with 576/1,025 cells (56.2%) having ≥1 field included on either the outbound or inbound rate maps. We then characterized the relationship between normalized location within the firing field, according to the decoded trajectory, and ripple band firing phase of the corresponding place cell using circular-linear regression. Remarkably, we found that the slope of this relationship was consistently negative and significantly different from zero across cells (Figure 4A). In addition, the distribution of within-field location versus ripple band firing phase intercepts was non-uniformly distributed, with a circular median value that was significantly later in the ripple cycle than the mean firing phase of all spikes fired (Figure 4B). As such, place cell firing typically shifted from late to early ripple band phases as replay trajectories passed through the firing field (Figure 4C).

Importantly, there was no difference in within-field location versus ripple band firing phase slopes between fields on the outbound and inbound maps, for cells with fields included on both maps, or by forward or reverse trajectories through these fields, for cells with sufficient spikes fired in each decoded movement direction through the same field (Figure S4B). Similarly, there was no difference in slopes between events in which each place cell fired only a single spike and those in which each place cell fired ≥3 spikes, across cells with sufficient in-field spikes in both cases (Figure S4C; see Figure 4C for an example). This indicates that, even when a place cell fired only a single spike within its firing field during a significant replay trajectory, it did so at a ripple band phase that indicated location within that firing field. Moreover, when analyzed separately, a negative relationship between decoded location within the place field and ripple band firing phase was observed across cells in all but one animal (range of −1.31 to 0.16 rad/field; $t(5) = -2.99$; $p < 0.05$). Quantitatively similar results were observed during online replay events, which occurred while the animal was awake but immobile at the corners of the track, suggesting that this phenomenon is preserved across behavioral states (Figure S5A). However, no such relationship was observed across putative excitatory cells in the deeper layers of MEC or across grid cells in that region specifically (Figures S5B and S5C).

To further characterize this phenomenon, we compared the properties of cells that exhibited negative versus positive within-field location versus ripple band phase slopes (n = 580

and n = 298 cells, respectively). However, we found no difference in the number of place fields expressed by those cells, average size of those place fields, average number of spikes fired in each place field, or peak in-field firing rates (all $p > 0.15$). In addition, we examined whether these results differed between cells that were recorded on the same tetrode as the LFP signal (n = 59) and those recorded on a different tetrode (n = 966) but found no difference in the slope or intercept of the within-field location versus ripple band firing phase relationship between groups (both $p > 0.09$). Finally, we asked whether these results would be affected by using a different, local LFP signal, where one was available, but found no change in the slope or intercept of the within-field location versus ripple band firing phase relationship across 47/63 cells that had ≥ 1 firing field with sufficient activity to include in this analysis (both $p > 0.12$).

Next, for comparison, we sought to characterize the relationship between within-field location and theta phase during movement on the track. To do so, we identified a total of 1,355/1,499 place fields on outbound and 1,256/1,423 place fields on inbound runs along the track that passed our criteria for inclusion (≥ 5 spikes fired within the field on all runs through that field, covering $\geq 50\%$ of the place field), with 873/1,044 (83.6%) cells having ≥ 1 field included on either outbound or inbound runs along the track. We then characterized the relationship between normalized location within the firing field (collapsed across all runs through the field in each session) and theta firing phase using circular-linear regression. Again, given that place cells tended to fire >1 spike during each oscillatory cycle, unlike during replay, we restricted these analyses to the first spike from each theta cycle. As expected, the slope of this relationship was consistently negative and significantly different from zero across cells (Figure 4D), with no difference in slopes between the outbound and inbound maps (across cells that had ≥ 1 field during runs in each direction; Figure S4D). In addition, the distribution of within-field location versus theta firing phase intercepts was non-uniform, with a circular median value that was significantly later in the theta cycle than the phase of peak firing (Figure 4E). As such, place cell firing typically shifted from late to early theta phases as the firing field was traversed (Figure 4F). Conversely, no such relationship was observed across putative excitatory cells in the deeper layers of MEC or in grid cells specifically (Figures S5D and S5E).

Finally, we directly compared within-field location versus phase relationships between ripple band oscillations during REST and theta oscillations during RUN, respectively, across n = 561 cells with ≥ 1 place field that passed our criteria for inclusion in each session. In this case (unlike the correlation in within-event time versus phase relationships between replay events during REST and movement-related n-spike trains during RUN, across cells), we found no correlation between within-field location versus phase slopes aggregated over multiple trajectories through each place field during RUN and REST ($r = -0.0399$; $p = 0.346$). However, we also found no evidence for any correlation in within-field location versus phase slopes between firing fields of the same cell on outbound and inbound runs during either REST or RUN or between ripple band phase precession slopes between forward and reverse trajectories through the same firing fields during REST (all $p > 0.19$). In addition, we found no evidence for a sub-population of place cells that consistently display positive within-field location versus firing phase slopes relative to both ripple oscillations during REST and theta oscillations during RUN ($\chi^2 = 1.59$; $p = 0.21$). This suggests that there is no relationship between within-field location versus phase slopes across cells between oscillation frequencies and behavioral states, between firing fields of the same cell, or between different movement directions during replay.

Coordinated phase precession across place cells generates "theta sweeps" of activity during movement, with place cells encoding for locations behind the animal being active early and place cells encoding for locations ahead of the animal being active later in each theta cycle.[7–9,33–35] To examine this phenomenon in our data, we split each theta cycle into ten discrete phase bins and computed the relative distance between the animal's average location in that cycle and the peak of the closest place field for all cells that were active in each phase bin (again, including only the first spike fired by each cell in each cycle). To facilitate subsequent comparison with replay events, we then averaged those data across all theta cycles in each continuous period of movement (defined as running speed ≥ 10 cm/s for a duration of ≥ 1 s) and used linear regression to estimate the distance covered by that average theta sweep (see STAR Methods for further details). This revealed an average forward sweep covering 13.2 ± 51.4 cm within each theta cycle, which was significantly different from zero across events, with an equivalent movement speed of 108 ± 504 cm/s, which significantly exceeds the animal's actual movement speed during the same events (Figure 5A). Crucially, theta sweeps also exhibit a positive slope (i.e., consistent with movement direction) in 68.5% of all events (more than expected by chance; binomial test; $p < 0.001$), indicating that they reliably encoded the animal's current trajectory.

Next, we asked whether coordinated ripple band phase precession across place cells during offline replay events also gave rise to ripple band sweeps of activity within each oscillatory cycle, analogous to the theta sweeps observed during active movement. To address this, we split each ripple band cycle into five discrete phase bins and computed the relative distance between the animal's average decoded location in that cycle and the peak of the closest place field for all cells that were active in each phase bin. We then averaged these data across all ripple band cycles in each significant replay event and used linear regression to estimate the distance covered by that average ripple band sweep. This revealed an average forward sweep covering 2.61 ± 56.3 cm within each ripple cycle, which was significantly different from zero across events (Figure 5B). In addition, the sequence of locations encoded by place cell firing across phase bins was consistent with movement direction of the decoded replay trajectory in 54.1% of all events, indicating that ripple band phase coding could be used to infer movement direction more often than expected by chance (binomial test; $p < 0.001$).[11,12] Interestingly, however, these forward sweeps exhibited an equivalent movement speed of 6.02 ± 134 m/s, which is not significantly different than decoded movement speed during the same events. Unlike theta sweeps, therefore, ripple band sweeps do not appear to have a strong prospective component.

DISCUSSION

We have demonstrated that the phase code for location exhibited by place cells during movement-related theta oscillations

is preserved during ripple band activity, when the hippocampus is believed to replay information to the neocortex. Specifically, we have shown that place cells that fire multiple spikes during candidate replay events do so at progressively earlier phases of the ongoing ripple band oscillation, that there is a consistently negative relationship between decoded location within the place field and ripple band firing phase across all replay trajectories that pass through the field, and that this is associated with forward sweeps of activity within each ripple cycle, which could be used to infer movement direction at the population level. Importantly, these results appear to be consistent across online and offline replay events (i.e., those recorded during quiescent waking periods on the track and during subsequent rest, respectively), suggesting that they are not dependent on behavioral state. In addition, the relationship between decoded within-field location and ripple band phase is consistent across events where cells fire only one spike or multiple spikes, indicating that it does not simply arise from changes in ripple band firing phase across multiple spikes within single events. In sum, this demonstrates that hippocampal phase coding is not restricted to place cell firing during active movement or to sustained low-frequency oscillations with relatively constant rhythmicity, consistent with a growing body of experimental work across species.[12,15–18]

These findings have two major implications for our understanding of neural coding and the function of the mammalian hippocampus. First, they suggest shared or similar mechanisms for updating place-cell activity according to real and simulated movement trajectories during theta and ripple band oscillations, respectively. Specifically, the phase coding mechanism hypothesized to update place cell activity during replay[36–38] might be the same as that used to update place cell activity according to path integration during actual movement.[39,40] Consistent with a relationship between theta and ripple band phase precession, we found that the slope of within-event time versus phase relationships during individual candidate replay events and movement-related n-spike trains were correlated across cells. Interestingly, however, the LFP phase of peak firing differs by $\sim\pi$ rad between theta and ripple band oscillations, potentially suggesting a different spatial distribution of LFP sources with a common mode of phase coding. Second, the phase coding of external and/or internal variables may reflect a more general mechanism for transmitting information from the hippocampus to downstream circuits. The theta phase code for distance traveled within the firing field improves the accuracy of location decoding[10] and allows movement direction to be inferred from population activity in each oscillatory cycle, which is not always possible using head direction cells.[11,12,29–31] More generally, the multiplexing of information in firing rate and phase could allow for much richer coding of task-relevant variables in the human brain across a range of network states and cognitive domains.[18,41–44]

Interestingly, we found no evidence for consistent changes in theta or ripple band firing phase in putative excitatory neurons recorded simultaneously in the deeper layers of MEC or across a sub-population of grid cells specifically. This suggests that ripple band phase precession may only be observed in cells that also exhibit theta phase precession. Future work might therefore look for ripple band phase precession in grid cells from the superficial layers of MEC, which are known to display robust theta phase precession and engage in replay events.[6,23,45] It has yet to be established whether large populations of grid cells in the deeper layers of MEC exhibit theta phase precession on linear tracks,[6] although previous studies have described this phenomenon in open field recordings,[46,47] in contrast to our results on the Z-shaped track. Elsewhere, recent studies have demonstrated a link between theta sweeps during active exploration and enhanced replay of trajectories through that environment.[36,48–50] Hence, future work should examine the development with experience of coordinated theta phase precession across place cells during active exploration and ripple band phase precession during subsequent replay trajectories. Given that we found equivalent ripple band phase precession during forward and reverse replay events, despite the fact that place cell firing has never occurred during reverse movement through the corresponding place field, this work could specifically investigate the relationship between the retrospective component of theta sweeps during active movement and subsequent ripple band phase precession during reverse replay events.[35]

One important question raised by these findings is whether downstream targets of the hippocampus could feasibly recover information encoded in ripple band firing phase. The duration of ripple band oscillatory cycles is on the order of $\sim$5 ms, and the phase range of ripple band precession is significantly less than that observed during theta oscillations, on the order of milliseconds and similar to the spike width of a typical pyramidal cell. It is not clear whether downstream neurons can distinguish inputs on a timescale of milliseconds, although we note that this is close to the timescale of integration in a typical cortical neuron, that coincidence detection may be facilitated by the large number of hippocampal neurons that are active in each candidate replay event ($\sim$25% on average), and that cortical dendrites are capable of distinguishing temporal input patterns on a similar timescale.[51] This question could be addressed in the future by computational modeling or by identifying methods for selectively disrupting the ripple band phase code and observing the effects on behavior.[52] In addition, future work should explore the differences between theta and ripple band forward sweeps—specifically, that the former consistently exhibit a strong prospective component although the latter do not. The encoded direction and speed of ripple band sweeps described here is highly variable, and although some of this variability is undoubtedly due to sampling error, it is also possible that the properties of ripple band sweeps are modulated by behavioral variables.[24]

In summary, we have demonstrated that place cells exhibit a ripple band phase code for location during replay, similar to the theta phase precession observed during movement. Consistent with a growing body of research, these findings suggest that the hippocampus might employ similar mechanisms to generate and project phase-coded information to downstream targets across a range of behavioral states.

STAR★METHODS

Detailed methods are provided in the online version of this paper and include the following:

- KEY RESOURCES TABLE
- RESOURCE AVAILABILITY

- Lead contact
- Materials availability
- Data and code availability
- EXPERIMENTAL MODEL AND SUBJECT DETAILS
- METHOD DETAILS
  - Recording
  - Behavioral Protocol
- QUANTIFICATION AND STATISTICAL ANALYSIS
  - Identifying Putative Principal Cells
  - Identifying Candidate Replay Events
  - Within-Event Time versus Ripple Firing Phase
  - Burst Index
  - Within-Event Time versus Theta Firing Phase
  - Generating Firing Rate Maps
  - Decoding Location on the Track
  - Decoding Location during Replay Events
  - Within-Field Location versus Ripple Firing Phase
  - Within-Field Location versus Theta Firing Phase
  - Theta and Ripple Band Sweeps

SUPPLEMENTAL INFORMATION

Supplemental information can be found online at https://doi.org/10.1016/j.cub.2021.10.033.

ACKNOWLEDGMENTS

The authors wish to thank Robin Hayman for his input during the preparation of this manuscript. H.F.O. is supported by a Donders Mohrmann fellowship, and this research was also funded in part by the Wellcome Trust (grant number 202805/Z/16/Z to N.B. and 212281/Z/18/Z to C.B.). For the purpose of open access, the author has applied a CC BY public copyright licence to any Author Accepted Manuscript version arising from this submission.

AUTHOR CONTRIBUTIONS

Project administration, D.B.; supervision, N.B. and C.B.; writing – original draft preparation, D.B.; writing – review and editing, D.B., H.F.O., C.B., and N.B.; investigation, H.F.O. and C.B.; methodology, D.B., H.F.O., C.B., and N.B.; funding acquisition, H.F.O., C.B., and N.B.

DECLARATION OF INTERESTS

The authors declare no competing interests.

Received: April 23, 2021
Revised: September 6, 2021
Accepted: October 14, 2021
Published: November 2, 2021

REFERENCES

1. O'Keefe, J., and Nadel, L. (1978). The Hippocampus as a Cognitive Map (Oxford University).
2. Eichenbaum, H. (2000). A cortical-hippocampal system for declarative memory. Nat. Rev. Neurosci. *1*, 41–50.
3. O'Keefe, J., and Dostrovsky, J. (1971). The hippocampus as a spatial map. Preliminary evidence from unit activity in the freely-moving rat. Brain Res. *34*, 171–175.
4. Hafting, T., Fyhn, M., Molden, S., Moser, M.B., and Moser, E.I. (2005). Microstructure of a spatial map in the entorhinal cortex. Nature *436*, 801–806.
5. O'Keefe, J., and Recce, M.L. (1993). Phase relationship between hippocampal place units and the EEG theta rhythm. Hippocampus *3*, 317–330.
6. Hafting, T., Fyhn, M., Bonnevie, T., Moser, M.B., and Moser, E.I. (2008). Hippocampus-independent phase precession in entorhinal grid cells. Nature *453*, 1248–1252.
7. Burgess, N., Recce, M., and O'Keefe, J. (1994). A model of hippocampal function. Neural Netw. *7*, 1065–1081.
8. Skaggs, W.E., McNaughton, B.L., Wilson, M.A., and Barnes, C.A. (1996). Theta phase precession in hippocampal neuronal populations and the compression of temporal sequences. Hippocampus *6*, 149–172.
9. Johnson, A., and Redish, A.D. (2007). Neural ensembles in CA3 transiently encode paths forward of the animal at a decision point. J. Neurosci. *27*, 12176–12189.
10. Jensen, O., and Lisman, J.E. (2000). Position reconstruction from an ensemble of hippocampal place cells: contribution of theta phase coding. J. Neurophysiol. *83*, 2602–2609.
11. Zutshi, I., Leutgeb, J.K., and Leutgeb, S. (2017). Theta sequences of grid cell populations can provide a movement-direction signal. Curr. Opin. Behav. Sci. *17*, 147–154.
12. Bush, D., and Burgess, N. (2020). Advantages and detection of phase coding in the absence of rhythmicity. Hippocampus *30*, 745–762.
13. Fries, P., Nikolić, D., and Singer, W. (2007). The gamma cycle. Trends Neurosci. *30*, 309–316.
14. Senior, T.J., Huxter, J.R., Allen, K., O'Neill, J., and Csicsvari, J. (2008). Gamma oscillatory firing reveals distinct populations of pyramidal cells in the CA1 region of the hippocampus. J. Neurosci. *28*, 2274–2286.
15. Zheng, C., Bieri, K.W., Hsiao, Y.-T., and Colgin, L.L. (2016). Spatial sequence coding differs during slow and fast gamma rhythms in the hippocampus. Neuron *89*, 398–408.
16. Jacobs, J. (2013). Hippocampal theta oscillations are slower in humans than in rodents: implications for models of spatial navigation and memory. Philos. Trans. R. Soc. Lond. B Biol. Sci. *369*, 20130304.
17. Eliav, T., Geva-Sagiv, M., Yartsev, M.M., Finkelstein, A., Rubin, A., Las, L., and Ulanovsky, N. (2018). Nonoscillatory phase coding and synchronization in the bat hippocampal formation. Cell *175*, 1119–1130.e15.
18. Qasim, S.E., Fried, I., and Jacobs, J. (2021). Phase precession in the human hippocampus and entorhinal cortex. Cell *184*, 3242–3255.e10.
19. Buzsáki, G., Horváth, Z., Urioste, R., Hetke, J., and Wise, K. (1992). High-frequency network oscillation in the hippocampus. Science *256*, 1025–1027.
20. Wilson, M.A., and McNaughton, B.L. (1994). Reactivation of hippocampal ensemble memories during sleep. Science *265*, 676–679.
21. O'Neill, J., Pleydell-Bouverie, B., Dupret, D., and Csicsvari, J. (2010). Play it again: reactivation of waking experience and memory. Trends Neurosci. *33*, 220–229.
22. Ólafsdóttir, H.F., Bush, D., and Barry, C. (2018). The role of hippocampal replay in memory and planning. Curr. Biol. *28*, R37–R50.
23. Ólafsdóttir, H.F., Carpenter, F., and Barry, C. (2016). Coordinated grid and place cell replay during rest. Nat. Neurosci. *19*, 792–794.
24. Ólafsdóttir, H.F., Carpenter, F., and Barry, C. (2017). Task demands predict a dynamic switch in the content of awake hippocampal replay. Neuron *96*, 925–935.e6.
25. Mizuseki, K., Sirota, A., Pastalkova, E., and Buzsáki, G. (2009). Theta oscillations provide temporal windows for local circuit computation in the entorhinal-hippocampal loop. Neuron *64*, 267–280.
26. Kempter, R., Leibold, C., Buzsáki, G., Diba, K., and Schmidt, R. (2012). Quantifying circular-linear associations: hippocampal phase precession. J. Neurosci. Methods *207*, 113–124.
27. Aghajan, Z.M., Acharya, L., Moore, J.J., Cushman, J.D., Vuong, C., and Mehta, M.R. (2015). Impaired spatial selectivity and intact phase precession in two-dimensional virtual reality. Nat. Neurosci. *18*, 121–128.
28. Huxter, J., Burgess, N., and O'Keefe, J. (2003). Independent rate and temporal coding in hippocampal pyramidal cells. Nature *425*, 828–832.

29. Cei, A., Girardeau, G., Drieu, C., Kanbi, K.E., and Zugaro, M. (2014). Reversed theta sequences of hippocampal cell assemblies during backward travel. Nat. Neurosci. *17*, 719–724.

30. Maurer, A.P., Lester, A.W., Burke, S.N., Ferng, J.J., and Barnes, C.A. (2014). Back to the future: preserved hippocampal network activity during reverse ambulation. J. Neurosci. *34*, 15022–15031.

31. Raudies, F., Brandon, M.P., Chapman, G.W., and Hasselmo, M.E. (2015). Head direction is coded more strongly than movement direction in a population of entorhinal neurons. Brain Res. *1621*, 355–367.

32. Ólafsdóttir, H.F., Barry, C., Saleem, A.B., Hassabis, D., and Spiers, H.J. (2015). Hippocampal place cells construct reward related sequences through unexplored space. eLife *4*, e06063.

33. Feng, T., Silva, D., and Foster, D.J. (2015). Dissociation between the experience-dependent development of hippocampal theta sequences and single-trial phase precession. J. Neurosci. *35*, 4890–4902.

34. Kay, K., Chung, J.E., Sosa, M., Schor, J.S., Karlsson, M.P., Larkin, M.C., Liu, D.F., and Frank, L.M. (2020). Constant sub-second cycling between representations of possible futures in the hippocampus. Cell *180*, 552–567.e25.

35. Wang, M., Foster, D.J., and Pfeiffer, B.E. (2020). Alternating sequences of future and past behavior encoded within hippocampal theta oscillations. Science *370*, 247–250.

36. Hasselmo, M.E. (2009). A model of episodic memory: mental time travel along encoded trajectories using grid cells. Neurobiol. Learn. Mem. *92*, 559–573.

37. Bicanski, A., and Burgess, N. (2018). A neural-level model of spatial memory and imagery. eLife *7*, e33752.

38. Yu, C., Behrens, T.E.J., and Burgess, N. (2020). Prediction with directed transitions: complex eigenstructure, grid cells and phase coding. arXiv, arXiv:2006.03355v1. https://arxiv.org/abs/2006.03355v1.

39. Mehta, M.R., Lee, A.K., and Wilson, M.A. (2002). Role of experience and oscillations in transforming a rate code into a temporal code. Nature *417*, 741–746.

40. Burgess, N., Barry, C., and O'Keefe, J. (2007). An oscillatory interference model of grid cell firing. Hippocampus *17*, 801–812.

41. Kurth-Nelson, Z., Economides, M., Dolan, R.J., and Dayan, P. (2016). Fast sequences of non-spatial state representations in humans. Neuron *91*, 194–204.

42. Heusser, A.C., Poeppel, D., Ezzyat, Y., and Davachi, L. (2016). Episodic sequence memory is supported by a theta-gamma phase code. Nat. Neurosci. *19*, 1374–1380.

43. Liu, Y., Dolan, R.J., Kurth-Nelson, Z., and Behrens, T.E.J. (2019). Human replay spontaneously reorganizes experience. Cell *178*, 640–652.e14.

44. Vaz, A.P., Wittig, J.H., Jr., Inati, S.K., and Zaghloul, K.A. (2020). Replay of cortical spiking sequences during human memory retrieval. Science *367*, 1131–1134.

45. O'Neill, J., Boccara, C.N., Stella, F., Schoenenberger, P., and Csicsvari, J. (2017). Superficial layers of the medial entorhinal cortex replay independently of the hippocampus. Science *355*, 184–188.

46. Climer, J.R., Newman, E.L., and Hasselmo, M.E. (2013). Phase coding by grid cells in unconstrained environments: two-dimensional phase precession. Eur. J. Neurosci. *38*, 2526–2541.

47. Jeewajee, A., Barry, C., Douchamps, V., Manson, D., Lever, C., and Burgess, N. (2013). Theta phase precession of grid and place cell firing in open environments. Philos. Trans. R. Soc. Lond. B Biol. Sci. *369*, 20120532.

48. Drieu, C., Todorova, R., and Zugaro, M. (2018). Nested sequences of hippocampal assemblies during behavior support subsequent sleep replay. Science *362*, 675–679.

49. Farooq, U., and Dragoi, G. (2019). Emergence of preconfigured and plastic time-compressed sequences in early postnatal development. Science *363*, 168–173.

50. Muessig, L., Lasek, M., Varsavsky, I., Cacucci, F., and Wills, T.J. (2019). Coordinated emergence of hippocampal replay and theta sequences during post-natal development. Curr. Biol. *29*, 834–840.e4.

51. Branco, T., Clark, B.A., and Häusser, M. (2010). Dendritic discrimination of temporal input sequences in cortical neurons. Science *329*, 1671–1675.

52. Robbe, D., and Buzsáki, G. (2009). Alteration of theta timescale dynamics of hippocampal place cells by a cannabinoid is associated with memory impairment. J. Neurosci. *29*, 12597–12605.

53. Barry, C., Hayman, R., Burgess, N., and Jeffery, K.J. (2007). Experience-dependent rescaling of entorhinal grids. Nat. Neurosci. *10*, 682–684.

54. Berens, P. (2009). CircStat: a MATLAB toolbox for circular statistics. J. Stat. Softw. *31*, 1–21.

55. Wills, T.J., Cacucci, F., Burgess, N., and O'Keefe, J. (2010). Development of the hippocampal cognitive map in preweanling rats. Science *328*, 1573–1576.

56. Sargolini, F., Fyhn, M., Hafting, T., McNaughton, B.L., Witter, M.P., Moser, M.B., and Moser, E.I. (2006). Conjunctive representation of position, direction, and velocity in entorhinal cortex. Science *312*, 758–762.

57. Langston, R.F., Ainge, J.A., Couey, J.J., Canto, C.B., Bjerknes, T.L., Witter, M.P., Moser, E.I., and Moser, M.B. (2010). Development of the spatial representation system in the rat. Science *328*, 1576–1580.

58. Zhang, K., Ginzburg, I., McNaughton, B.L., and Sejnowski, T.J. (1998). Interpreting neuronal population activity by reconstruction: unified framework with application to hippocampal place cells. J. Neurophysiol. *79*, 1017–1044.

59. Davidson, T.J., Kloosterman, F., and Wilson, M.A. (2009). Hippocampal replay of extended experience. Neuron *63*, 497–507.

STAR★METHODS

KEY RESOURCES TABLE

REAGENT or RESOURCE	SOURCE	IDENTIFIER
Deposited data		
Dataset used in the present study	Previously published and described[23]	https://zenodo.org/record/5566548
Experimental models: organisms/strains		
Lister Hooded rats	Charles River	RRID:RGD_2312466, https://www.criver.com/products-services/basic-research/find-a-model/lister-hooded
Software and algorithms		
MATLAB	Mathworks, MA	RRID: SCR_001622, https://uk.mathworks.com/products/matlab.html
Tint spike sorting software	Axona	Product code: COMP/TINT01, http://axona.com/products
Other		
Recording system (pre-amp and systems unit)	Axona	Product code: Dacq/USB64, http://axona.com/products
Omnetic connectors (microdrive assembly)	Genalog	Product code: A79026-001, https://genalog.com/genaloglinecard/omnetics/
Single-screw mouse microdrive	Axona	Product code: MDMR-01M1, http://axona.com/products
4 × 16 channel headstage preamplifiers	Axona	Product code: HS-116M1D, http://axona.com/products
Microwire (17um, platinum iridium)	California Fine Wire Company	Product code: 100167, https://www.calfinewire.com/datasheets/100167-platinum10iridium.html
NanoZ plating equipment	Multichannel Systems	nanoZ, https://www.multichannelsystems.com/products/nanoz
4 x fine wire tethers	Axona	Product code: HS16-CAB3, http://axona.com/products

RESOURCE AVAILABILITY

Lead contact

Further information and requests for resources should be directed to and will be fulfilled by the Lead Contact, Daniel Bush (drdanielbush@gmail.com)

Materials availability

This study did not generate new unique reagents

Data and code availability

- All data analyzed in this study are publicly available from Zenodo (https://zenodo.org/record/5566548)
- This paper does not report original code
- Any additional information required to reanalyse the data reported in this paper is available from the lead contact upon request

EXPERIMENTAL MODEL AND SUBJECT DETAILS

Six male Lister Hooded rats (330–400 g at implantation) received two microdrives, each carrying eight tetrodes of twisted 17 μm HM-L coated platinum iridium wire (90% and 10%, respectively; California Fine Wire), targeted to right CA1 (ML: 2.2 mm, AP: 3.8 mm posterior to bregma) and left medial entorhinal cortex (MEC; ML = 4.5 mm, AP = 0.3–0.7 anterior to the transverse sinus, angled between 8–10°). Wires were platinum plated to reduce impedance to 200–300 k at 1 kHz. After rats had recovered from surgery, they were maintained at 90% of free-feeding weight with *ad libitum* access to water and were housed individually on a

CellPress
OPEN ACCESS

12-h light-dark cycle. All procedures were approved by the UK Home Office, subject to the restrictions and provisions contained in the Animals (Scientific Procedures) Act of 1986.

METHOD DETAILS

Recording

Screening was performed post-surgically after a 1-week recovery period. An Axona recording system (Axona Ltd.) was used to acquire single units and position data (for details of the recording system and basic recording protocol see Barry et al.[53]). Position and head direction were inferred using an overhead video camera to record the location of two light-emitting diodes (LED) mounted on the animals' head-stages at a sample rate of 50 Hz. Tetrodes were gradually advanced in 62.5 μm steps across days until place cells (CA1) or grid cells (MEC) were found. EEG data were concurrently acquired at a sample rate of 4800Hz.

Behavioral Protocol

The experiment was run during the animals' light period, to facilitate rest during the REST session. During RUN sessions animals shuttled back and forward on a Z-shaped track comprised of 10cm wide runways covered with black paint, raised 75cm off the ground. The two parallel sections of the Z (190cm each) were connected by a diagonal section (220cm; see Figure 1A). The entire track was surrounded by plain black curtains. Animals were pre-trained to run on the track, taking between 3 and 6d before they would shuttle fluently from one end to the other. At the start of each session, rats were placed at one end of the Z-track. The same end was used as a starting location for every day of the experiment and for every rat. The ends and corners of the track were baited with sweetened rice to encourage running from one end to the other.

Following the RUN session, rats were placed in the REST enclosure for an hour and a half. The rest enclosure consisted of a cylindrically shaped environment (18cm diameter, 61cm high) with a towel placed at the bottom and was located outside of the curtains that surrounded the Z-track. Animals were not able to see the surrounding room while in the rest enclosure. Prior to recording, rats had been familiarized with the rest environment for at least 7d. Following the REST session, rats foraged in a familiar open field environment for 20 min, to allow functional classification of MEC cells[23].

QUANTIFICATION AND STATISTICAL ANALYSIS

All analysis was carried out using MATLAB (Mathworks, Natick MA) and all circular statistics were computed using CircStat: A MATLAB Toolbox for Circular Statistics[54]. All statistical results, including details of the tests used and degrees of freedom, are reported in the main text and corresponding figure legends.

Identifying Putative Principal Cells

All analyses were restricted to putative principal cells, identified by manual inspection of waveforms and mean firing rates $\leq$ 10Hz across the entire recording session. In addition to 1025 CA1 cells during REST and 1044 during RUN, we recorded a total of 877 cells in the deeper layers of MEC during REST, of which 832 were classed as putative principal cells (median ± SD = 31 ± 9.94 per session, range 12-46); and a total of 1112 cells during RUN, of which 1033 were classed as putative principal cells (37 ± 13.4 per session, range 15-82). We classified MEC cells as grid cells using a shuffling procedure similar to that applied elsewhere[55]. Specifically, we generated firing rate maps for each cell during the twenty minute foraging session that followed REST. We then computed both the standard[56] and modified[57] gridness measures for each cell and compared those values with a null distribution generated by randomly permuting the spike train of each cell relative to the tracking data by a temporal distance of $\geq$ 30 s, 100 times. Grid cells were subsequently classified as those for which the standard or modified gridness scores exceeded the 97.5th percentile of the matching null distribution. This identified 76/832 grid cells during REST and 80/1033 during RUN.

Identifying Candidate Replay Events

We used two methods to identify candidate replay events – either as periods of elevated multi-unit activity (MUA) or ripple band power. In the former case, we first generated a MUA histogram for all putative principal cells in 1ms time bins. This histogram was then smoothed using a Gaussian kernel with 5ms standard deviation and candidate replay events identified as periods of $Z \geq 0$ with peak $Z \geq 3$. Any candidate events separated by $\leq$ 40ms were merged and any remaining events with a duration of $\leq$ 40ms discarded. Finally, we excluded any events during which < 5 or 15% of all pyramidal cells were active (whichever is the larger), median running speed > 10cm/s, or duration > 0.5 s.

In the latter case, we first identified the EEG channel in each RUN session with the highest 6-12Hz theta signal to noise ratio. Next, we filtered LFP data from that channel in the 150-250Hz ripple band using a 400th order finite impulse response (FIR) filter, extracted the amplitude of the filtered signal using the Hilbert transform and smoothed that time series using a Gaussian kernel with 5ms standard deviation. Candidate replay events were then identified as periods with ripple band amplitude $Z \geq 0$ and peak ripple band amplitude $Z \geq 3$. Any candidate events separated by $\leq$ 40ms were merged and any remaining events with a duration of $\leq$ 40ms discarded. Finally, we excluded any events during which < 5 or 15% of all pyramidal cells were active (whichever is the larger), median running speed > 10cm/s, or duration > 0.5 s.

We generated wavelet spectrograms for each event using a five cycle Morlet wavelet transform in a [-0.2:0.2 s] window centered on the time of peak MUA. These spectrograms were log transformed and averaged across events before being baseline corrected using average power at each frequency in log transformed wavelet spectrograms of periods between candidate replay events. Specifically, we used the central 0.4 s section of the EEG signal between each pair of candidate replay events that were separated by $\geq$ 2.4 s (i.e., with $\geq$ 1 s padding that did not overlap with any candidate replay event).

Within-Event Time versus Ripple Firing Phase

To examine changes in the ripple band firing phase of active cells over the course of candidate replay events, we first identified the EEG channel in each RUN session with the highest 6-12Hz theta signal to noise ratio. Next, we filtered LFP data from that channel in the 150-250Hz ripple band using a 400th order FIR filter, extracted the phase of the filtered signal at each time point using the Hilbert transform, and computed the preferred ripple band firing phase of each cell as the circular mean firing phase across all candidate replay events. We then adjusted LFP and firing phase values such that the circular mean preferred firing phase across all putative principal cells in each session was equal to π rad. In addition, we quantified the ripple band phase modulation of firing for each cell as the resultant vector length of ripple band firing phases across all candidate replay events (referred to as the phase locking value, PLV). To establish the significance of phase modulation, we compared the true resultant vector length with a surrogate distribution generated by randomly permuting the spike train of each cell relative to LFP ripple band phase by a temporal distance of $\geq$ 30 s, 1000 times, and setting the threshold for significance as the 99th percentile of that distribution. For illustration purposes, we also generated circular firing rate histograms for each cell using 30 equally sized phase bins.

Next, we identified all cells in each candidate event that fired $\geq$ 3 spikes and computed both the circular mean phase shift between successive spikes; and the circular-linear correlation between normalized spike time (i.e., where the first spike fired by that cell in that replay event occurs at $t = 0$ and the last at $t = 1$) and ripple band firing phase (see Figure 1B for an illustration), for each candidate event[26]. The latter provides an intercept and slope of the circular-linear relationship between within-event time and ripple band phase relationship for that cell in that event. We subsequently computed the average phase shift, slope and circular mean intercept across all events for each cell. In addition, we repeated the analysis above but considered only the first spike fired in each oscillatory cycle, in which case only cells that fire in $\geq$ 3 cycles were included. Finally, we estimated phase auto-correlograms for each n-spike cell in each n-spike event from the temporal autocorrelation of unwrapped ripple band spike phases, averaged those across events for each cell and then across cells.

Burst Index

To ascertain whether ripple band phase shifts were driven by bursting, we quantified the ‘burst index’ of each cell as the relative proportion of ISIs in the $\leq$ 200ms range that were $\leq$ 10ms. We subsequently correlated burst indices with circular mean phase shifts (using the circular-linear correlation, with 1000 shuffles to establish significance) and mean within-event time versus ripple band phase slopes across cells.

Within-Event Time versus Theta Firing Phase

To examine changes in the theta firing phase of active cells over the course of movement related n-spike trains, we first generated a spike train histogram for each cell with a bin size of 5ms using only spikes fired at a running speed of $\geq$ 10cm/s. This histogram was smoothed using a Gaussian kernel with 80ms standard deviation, converted to a firing rate, and candidate theta spike trains identified as contiguous periods with a mean firing rate $\geq$ 1Hz. Any candidate events separated by $\leq$ 500ms were merged and any remaining events with duration < 500ms discarded. Finally, we excluded any events that incorporate < 3 spikes. Because these analyses included only the first spike fired in each theta cycle, this meant discarding all events in which cells fired in < 3 cycles.

Next, we filtered LFP data in the 6-12Hz theta band using a 400th order FIR filter, extracted the phase of the filtered signal at each time point using the Hilbert transform, and computed the preferred theta firing phase of each cell as the circular mean firing phase across all candidate theta spike trains. We then adjusted LFP and firing phase values such that the circular mean preferred firing phase across all putative principal cells in each session was equal to π rad.

In addition, we quantified the theta phase modulation of firing for each cell as the resultant vector length of theta firing phases across all spikes fired at a running speed of $\geq$ 10cm/s (again, referred to as the PLV). To establish the significance of phase modulation, we compared the true resultant vector length with a surrogate distribution generated by randomly permuting the spike train of each cell relative to LFP theta phase by a temporal distance of $\geq$ 30 s, 1000 times, and setting the threshold for significance as the 99th percentile of that distribution. For illustration purposes, we also generated circular firing rate histograms for each cell using 30 equally sized phase bins.

Finally, we computed both the circular mean phase difference between successive spikes in each theta spike train; and the circular-linear correlation between normalized spike time (i.e., where the first spike fired by that cell in that theta spike train occurs at $t = 0$ and the last at $t = 1$) and theta firing phase, including the first spike fired in each theta cycle only. This provided an intercept and slope for the circular-linear relationship between within-event time and theta phase for that cell in that event. We subsequently computed the average phase shift, slope and circular mean intercept across all events for each cell.

Generating Firing Rate Maps

To generate firing rate maps for each active cell in each session, we first excluded spikes fired in areas where the animals regularly performed non-perambulatory behaviors (for example, eating or grooming) - specifically, in the final 10 cm at either end of the track

and 5 cm around each of the two corners – and from 'unsuccessful' runs (i.e., those in which the animal did not progress from one end of the Z track, along all three sections to the other end). We then linearized each animal's path and computed dwell time and the total number of spikes fired at running speeds of $\geq$ 10cm/s in 2cm bins. These histograms were each smoothed using a Gaussian kernel with standard deviation of 5 bins, and firing rates computed by dividing the spike number by dwell time. We generated separate rate maps for runs in the outbound and inbound directions, and defined place fields for each cell as contiguous regions of at least 10 spatial bins with firing rate Z $\geq$ 0 and a peak in-field firing rate of $\geq$ 1Hz.

Decoding Location on the Track

Before decoding location during candidate replay events, we first used a Bayesian decoding framework to establish the accuracy with which location on the track could be decoded from activity across a population of N place cells during each RUN session[58]. To do so, we computed the number of spikes k fired by each cell i in consecutive T = 500ms time bins during successful outbound and inbound runs when the running speed of the animal was $\geq$ 10cm/s. Using the firing rate maps $\alpha_i(x)$ for each cell on outbound and inbound runs, respectively, we then computed a posterior probability distribution $P(x|K)$ describing the likelihood that the animal was located in each 2cm bin of the firing rate map, given the population spiking activity, according to Equation 1. The true location in each time bin was taken as the average location across all samples within that bin, and the decoded location was taken as the spatial bin with the greatest probability across all spatial bins. This allowed a decoding error to be computed for each time bin, and a median error across all time bins to be computed for outbound (13.7cm) and inbound (12.8cm) runs in each session, respectively. As expected, the median decoding error for each session (averaged across runs in each direction) was strongly negatively correlated with the total number of pyramidal cells recorded ($r = -0.65$, $p < 0.001$).

$$P(x|K) = \prod_{i=1}^{N} \frac{T\alpha_i(x)^{k_i}}{k_i!} exp^{-T\alpha_i(x)} \qquad \text{(Equation 1)}$$

Decoding Location during Replay Events

Next, we used similar methods to decode location from place cell population activity during candidate replay events. In that case, however, we computed the number of spikes k fired by each cell i in consecutive time bins of T = 10ms throughout the replay event, starting at the time of the first spike fired by any cell. We then decoded location independently in each time bin where at least one spike was fired by any cell using the Bayesian framework described by Equation 1 (note that this includes all putative pyramidal cells, whether or not they have a place field on the track in either running direction). This provides a spatial bin x time bin posterior probability matrix for each candidate event, with probability values in each time bin normalized so that they sum to one.

We then identified the linear fit thorough that posterior probability matrix which maximizes the summed probability under a line of fifteen spatial bins in width (i.e., 30cm), testing all potential spatial bins as a starting point and movement speeds of 100 to 5000 cm/s in steps of 50 cm/s, in either forward or reverse directions along the track[59]. We recorded the intercept (i.e., spatial origin of the decoded trajectory) and slope (i.e., running speed of the decoded trajectory) of that line, as well as the average summed probability per time bin (or 'fit score'). To establish the significance of each decoded trajectory, we shuffled the identity of active cells within each event 100 times (noting that our minimum threshold of 5 active cells allows a minimum of 120 unique shuffles) and re-computed the optimal fit score for each shuffle. An event is considered to be significant if the true fit score exceeds the 95th percentile of the fit score distribution obtained by shuffling. As expected, there is a strong positive correlation between the total number of pyramidal cells recorded in a session and the overall proportion of significant events ($r = 0.646$, $p < 0.001$).

Within-Field Location versus Ripple Firing Phase

To examine the relationship between decoded location within a firing field and ripple band firing phase, we first use the linear trajectories identified above to compute the average decoded location in each ripple band cycle in each significant replay event. We use these decoded locations to identify all spikes fired by each cell within each firing field across all significant replay events in each session. We then compute the circular-linear correlation between the relative distance of each spike through the field (given the decoded movement direction) and ripple band firing phase for any field with $\geq$ 5 spikes that cover $\geq$ 50% of the place field. This provided an intercept and slope for the circular-linear relationship between decoded within-field location and ripple band firing phase for each place field. We subsequently computed the average slope and circular mean intercept across all fields for each cell.

Within-Field Location versus Theta Firing Phase

To examine the relationship between actual location within a firing field and theta firing phase, we computed the circular-linear correlation between the relative distance traveled through the field and theta firing phase for any field with $\geq$ 5 spikes that covered $\geq$ 50% of the place field. Importantly, we included only the first spike fired in each theta cycle in this analysis. This provided an intercept and slope of the circular-linear relationship between within-field location and theta firing phase for each place field. We subsequently computed the average slope and circular mean intercept across all fields for each cell.

Theta and Ripple Band Sweeps

To examine theta sweeps during movement on the track, and facilitate comparison with individual replay events, we first identify 'movement events': periods of continuous movement with running speed $\geq$ 10cm/s and minimum duration $\geq$ 1 s. For each active cell in each movement event, we extract the theta phase at which the first spike was fired during each oscillatory cycle, and the relative distance between the average true location of the animal in that theta cycle and the peak of the nearest place field of that cell. Next, we compute the median relative distance across all spikes fired by all cells in all cycles within that event for ten equally sized theta phase bins, to provide an estimate of the average theta sweep for that event. Finally, we used linear regression to estimate the slope of the relationship between theta phase and decoded location, restricting our analyses to theta phase bins that showed forward movement in the grand average plot (Figure 5A, specifically, between 0 and $7\pi/5$ rad). These slopes were subsequently used to estimate the overall range and corresponding speed of theta sweeps for each event.

To examine ripple band sweeps during sleep, we extract the ripple band firing phase of all spikes fired by all active cells in each significant replay event and compute the relative distance between the average decoded location in that ripple band cycle (estimated using the slope and intercept of fitted trajectories) and the peak of the nearest place field of that cell. Next, we compute the median relative distance across all spikes fired by all cells in all cycles within that event, for five equally sized ripple band phase bins, to provide an estimate of the average ripple sweep for that event. Finally, we used linear regression to estimate the slope of the relationship between ripple band phase and decoded location, restricting our analyses to ripple band phase bins that showed forward movement in the grand average plot (Figure 5B, specifically, between $2\pi/5$ and 2π rad). These slopes could subsequently be used to estimate the overall range and corresponding speed of theta sweeps for each event.

Current Biology, Volume *32*

Supplemental Information

Ripple band phase precession of place cell firing during replay

Daniel Bush, H. Freyja Ólafsdóttir, Caswell Barry, and Neil Burgess

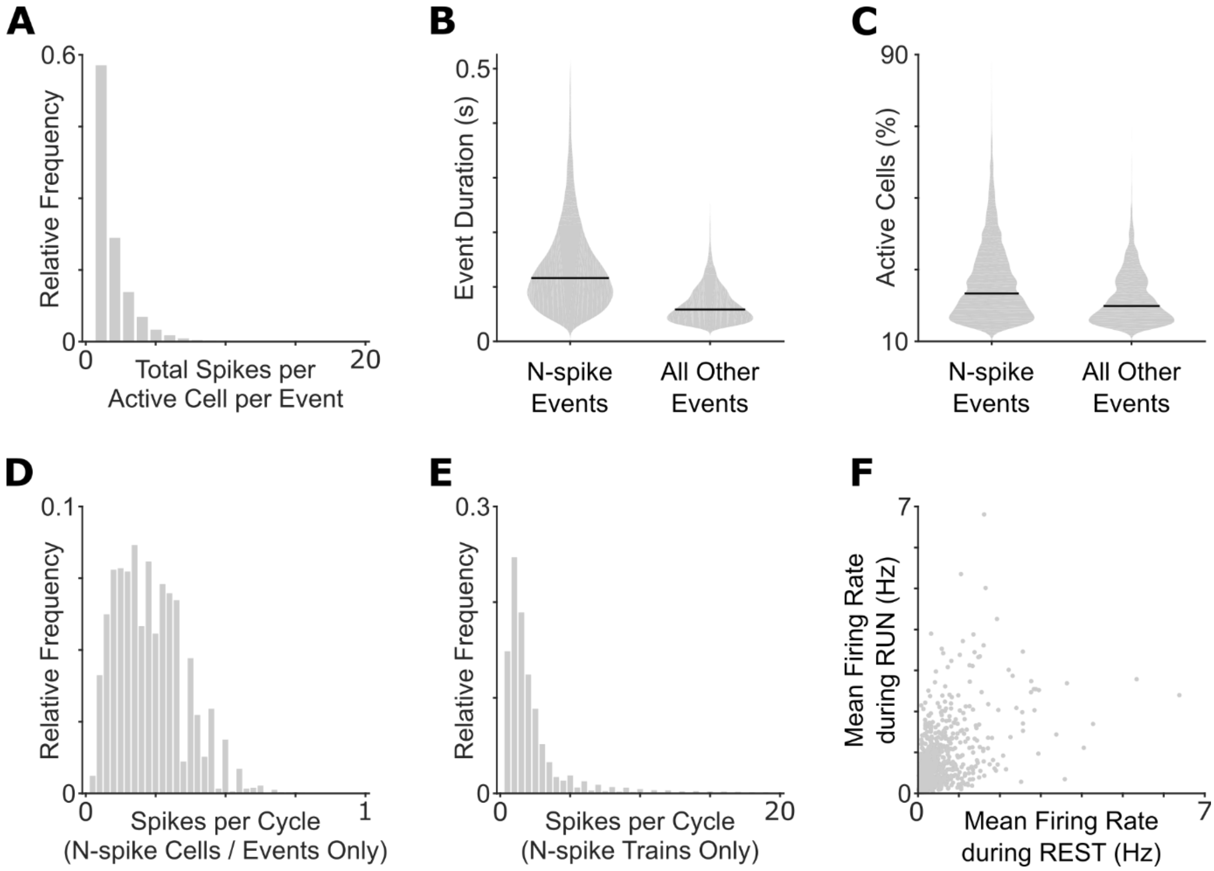

Figure S1: Properties of Candidate Replay Events and Movement Related N-spike Trains, Related to Figure 2

[A] Total number of spikes fired by active place cells within each candidate replay event detected from increased multi-unit activity (MUA) during REST (median±SD = 1±1.4)

[B] Duration of n-spike candidate replay events and all other events. N-spike events tend to be longer (127±72.7 vs. 71±30.9ms; Mann-Whitney U-test, Z = 70, p<0.001)

[C] Proportion of active place cells in n-spike candidate replay events and all other events. N-spike events tend to incorporate a greater proportion of active cells (24.1±10.6 vs. 20.7±7.17%; Mann-Whitney U-test, Z = 27.8, p<0.001)

[D] Spikes fired per ripple cycle by active n-spike cells in candidate replay events during REST (0.179±0.108)

[E] Spikes fired per theta cycle by active cells in movement related n-spike trains during RUN (1.57±3.0)

[F] Mean firing rates during REST and RUN are highly correlated across place cells (r = 0.507, p<0.001), although firing rates are significantly higher during RUN (median = 0.458 vs. 0.209Hz, t(959) = 11.9, p<0.001)

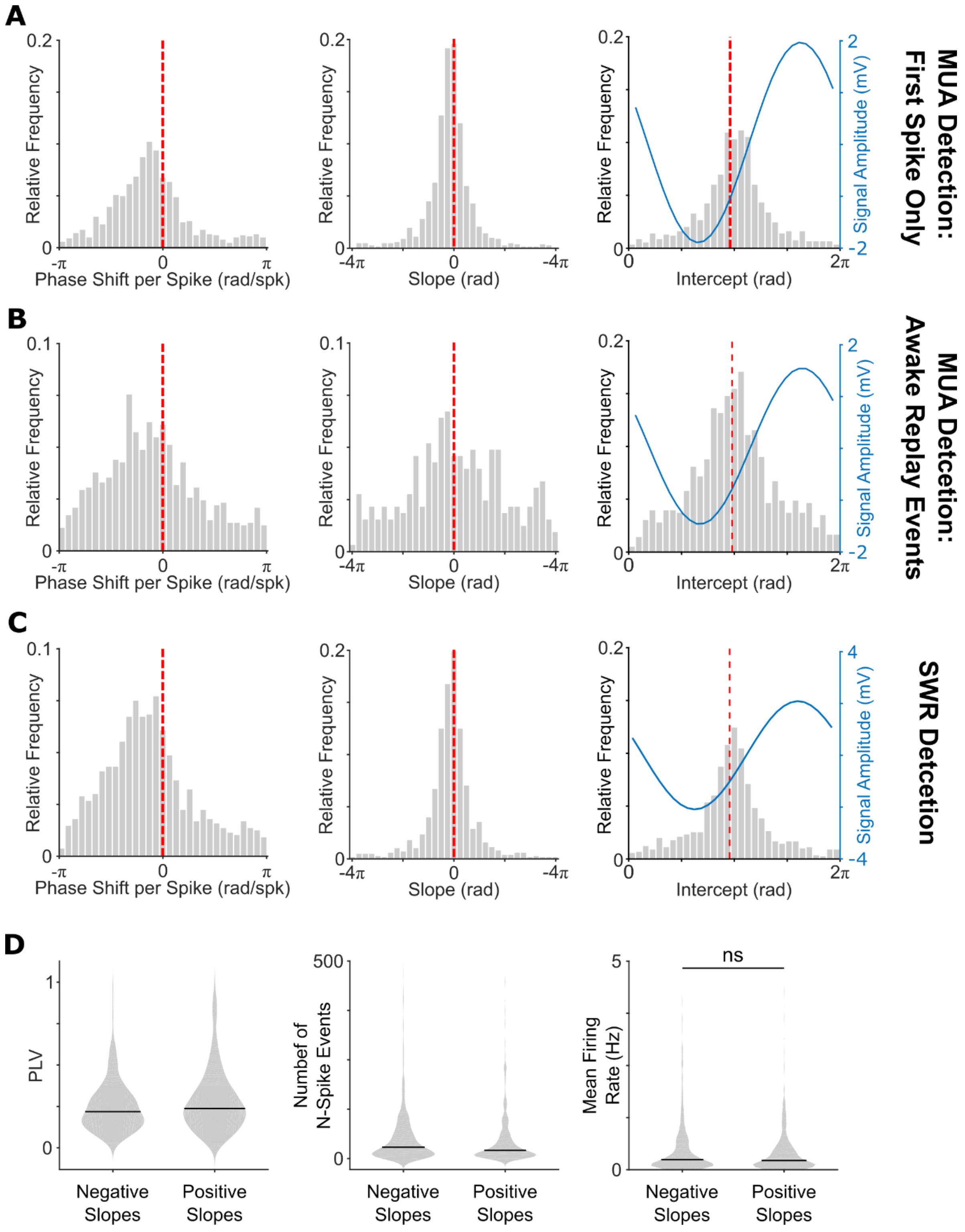
A
MUA Detection: First Spike Only
B
MUA Detection: Awake Replay Events
C
SWR Detection
D
Relative Frequency
Phase Shift per Spike (rad/spk)
Slope (rad)
Intercept (rad)
Signal Amplitude (mV)
PLV
Numbef of N-Spike Events
Mean Firing Rate (Hz)
Negative Slopes
Positive Slopes
ns

Figure S2: Phase Shifts and Within-Event Time vs. Ripple Band Phase Relationships across Behavioural States and Replay Detection Methods, Related to Figure 3

[A] Changes in place cell ripple band firing phase during offline candidate replay events detected from increased MUA, when only the first spike fired in each oscillatory cycle is included. **Left**: Circular mean phase shift between successive spikes across cells (n=951, overall circular median±circular SD=-0.553±1.13 rad/spk). This distribution is non-uniform (Rayleigh test, z=265, p<0.001) with a median value that differs from zero (circular median test, p<0.001); **Middle**: Distribution of within-event time vs. ripple band phase slopes across cells (overall median±SD=-0.459±2.74 rad), which differs from zero (t(947)=-5.91, p<0.001); **Right**: Distribution of intercepts across cells (overall circular mean±circular SD=3.14±1.0 rad). This distribution is non-uniform (Rayleigh test, z=348, p<0.001) with a median value that differs from the preferred firing phase of each cell (3.01±0.79 rad marked with a red dashed line, circular median test, p<0.001)

[B] Changes in place cell ripple band firing phase during online candidate replay events detected from increased MUA. We identified a total of 5590 online replay events using this method (median±SD=133±146 per session, range 62-593) lasting 117±64.4ms with 25±9.91% cells active per event each firing 1±1.56 spikes in total. **Left**: Circular mean phase shift between successive spikes across cells (-0.649±1.51 rad/spk). This distribution is non-uniform (z=84.3, p<0.001) with a median value that differs significantly from zero (p<0.001); **Middle**: Distribution of within-event time vs. ripple band phase slopes across cells (-0.299±3.80 rad), which differs significantly from zero (t(864)=-2.23, p<0.05); **Right**: Distribution of intercepts across cells (3.09±1.31 rad). This distribution is non-uniform (z=155, p<0.001), but the median value does not differ significantly from the preferred firing phase of each cell (3.08±1.08 rad, marked with a red dashed line)

[C] Changes in place cell ripple band firing phase during offline candidate replay events detected from increased ripple band power. We identified a total of 20151 offline replay events using this method (median±SD=686±411 per session, range 112-2175) lasting 155±90.2ms with 25.0±10.6% cells active per event each firing 1±1.31 spikes in total. **Left**: Circular mean phase shift between successive spikes across cells (-0.653±1.33 rad/spk). This distribution is non-uniform (Rayleigh test, z=158.8, p<0.001) with a median value that differs from zero (circular median test, p<0.001); **Middle**: Distribution of within-event time vs. ripple band phase slopes across cells (-0.354±2.91 rad), which differs from zero (t(916)=-4.45, p<0.001); **Right**: Distribution of intercepts across cells (3.03±1.05 rad). This distribution is non-uniform (Rayleigh test, z=303, p<0.001), but the median value does not differ significantly from the preferred firing phase of each cell (3.0±0.783 rad, marked with a red dashed line)

[D] Place **c**ells with negative within-event time v ripple band phase slopes during offline candidate replay events detected from increased MUA were less phase locked to ripple band oscillations (t(948)=2.78, p<0.01) and participated in a greater number of candidate replay events (t(948)=2.13, p<0.05) than cells with positive slopes, without any difference in overall mean firing rates (p=0.21)

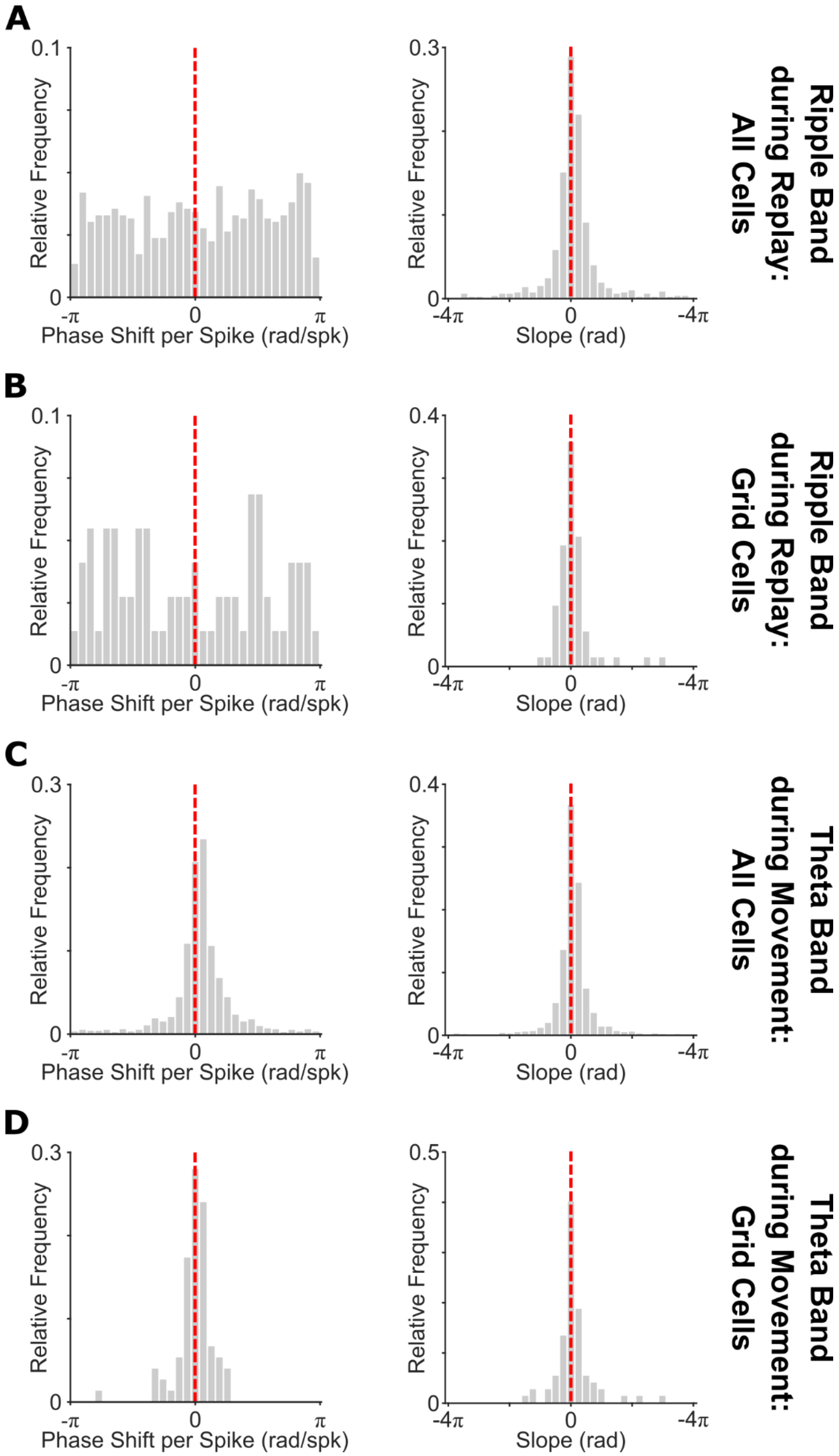
A
0.1
Relative Frequency
0
-π
0
π
Phase Shift per Spike (rad/spk)
0.3
Relative Frequency
0
-4π
0
-4π
Slope (rad)
Ripple Band during Replay: All Cells
B
0.1
Relative Frequency
0
-π
0
π
Phase Shift per Spike (rad/spk)
0.4
Relative Frequency
0
-4π
0
-4π
Slope (rad)
Ripple Band during Replay: Grid Cells
C
0.3
Relative Frequency
0
-π
0
π
Phase Shift per Spike (rad/spk)
0.4
Relative Frequency
0
-4π
0
-4π
Slope (rad)
Theta Band during Movement: All Cells
D
0.3
Relative Frequency
0
-π
0
π
Phase Shift per Spike (rad/spk)
0.5
Relative Frequency
0
-4π
0
-4π
Slope (rad)
Theta Band during Movement: Grid Cells

Figure S3: Phase Shifts and Within-Event Time vs. Phase Relationships in MEC, Related to Figures 2 and 3

[A] Changes in the ripple band firing phase of putative principal cells during offline candidate replay events detected from increased MUA in medial entorhinal cortex (MEC). We identified a total of 67540 offline replay events using this method (2701±1144 per session, range 268-4743) lasting 73±54.7ms with 25±9.96% cells active per event each firing 1±1.44 spikes in total. **Left**: Circular mean phase shift between successive spikes across 769/832 n-spike cells. This distribution is non-uniform (Rayleigh test, $z=3.02$, $p=0.05$) with a median value that does not differ from zero (circular median test, $p=0.15$), but does differ from the phase shift across place cells (Watson-Williams test, $F=215$, $p<0.001$); **Right**: Distribution of within-event time vs. ripple band phase slopes across cells (0.172±2.52 rad), which differs both from zero ($t(768)=2.63$, $p<0.01$) and from slopes across place cells ($t(1737)=-6.44$, $p<0.001$)

[B] Changes in the ripple band firing phase of grid cells during offline candidate replay events detected from increased MUA in MEC. **Left**: Circular mean phase shift between successive spikes across 73/832 n-spike grid cells. This distribution is uniform (Rayleigh test, $z=0.848$, $p=0.43$) and differs from the phase shift across place cells (Watson-Williams test, $F=26.5$, $p<0.001$); **Right**: Distribution of within-event time vs. ripple band phase slopes across cells (-0.119±1.83 rad), which is not different from zero ($t(72)=1.13$, $p=0.26$) but does differ from slopes across place cells ($t(1042)=-2.66$, $p<0.01$)

[C] Changes in the theta firing phase of putative principal cells during movement related n-spike trains in MEC. We identified a total of 121538 spike trains (4635±1936 per session, range 856-7218) lasting 1.53±1.37s and incorporating 5.0±6.66 spikes per train. **Left**: Circular mean phase shift between successive spikes across 1011/1033 n-spike cells (0.171±0.714 rad/spk). This distribution is non-uniform (Rayleigh test, $z=607$, $p<0.001$) with a median value that differs from zero (circular median test, $p<0.001$); **Right**: Distribution of within-event vs. theta phase slopes across cells (0.185±1.74 rad), which differs from zero ($t(1008)=3.99$, $p<0.001$)

[D] Changes in the theta firing phase of grid cells during movement related n-spike trains in MEC. **Left**: Circular mean phase shift between successive spikes across 75/1033 n-spike grid cells (0.0133±0.457 rad/spk). This distribution is non-uniform (Rayleigh test, $z=60.8$, $p<0.001$) with a median value that does not differ from zero (circular median test, $p=0.49$); **Right**: Distribution of within-event time vs. theta phase slopes across cells (0.119±2.02 rad), which is not different from zero ($t(74)=1.08$, $p=0.29$)

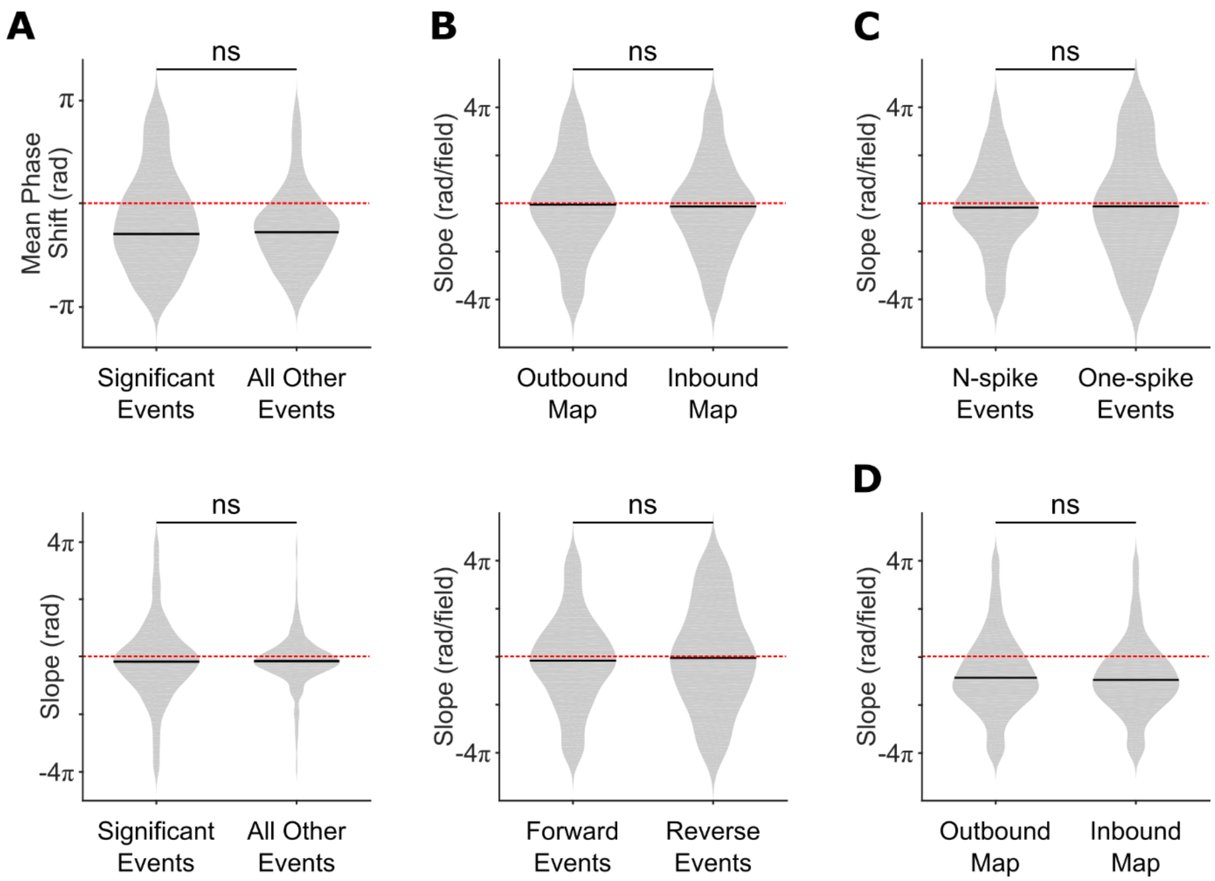

Figure S4: Comparing Phase Precession across Replay Events, Trajectory Directions and Firing Rate Maps, Related to Figures 3 and 4

[A] Comparison of within-event phase shift and time vs. ripple band phase slopes between significant and non-significant linear replay events. There was no difference in either phase shifts per spike pair (**upper panel:** circular median test, p=0.54; significant=-0.936±1.50, non-significant=-0.882±1.13 rad/spk) or slopes (**lower panel:** t(779)=-0.048, p=0.96; significant-0.559±4.30, non-significant=-0.506±2.37 rad) between these events across cells

[B] Comparison of within-field location vs. ripple band phase slopes between significant events on the outbound and inbound map; forward and reverse events. There was no difference in slopes between the outbound and inbound maps, for cells with fields included on both maps (**upper panel:** t(277)=-0.596, p=0.55; outbound=-0.201±6.09, inbound=-0.437±6.03 rad/field); or by forward or reverse trajectories, for cells with sufficient spikes fired in each decoded movement direction through the same field (**lower panel:** t(343)=-0.825, p=0.41; forward=-0.556±6.03, reverse=-0.201±6.07 rad/field)

[C] Comparison of within-field location vs. ripple band phase slopes between n-spike and one-spike events. There was no difference in slopes computed using events in which each place cell fired ≥3 spikes and events in which each place cell fired just one spike, across cells with sufficient in-field spikes in both cases (t(150)=0.158, p=0.88; n-spike events=-0.614±5.77, one-spike events=-0.478±6.90 rad/field)

[D] Comparison of within-field location vs. theta phase slopes between the outbound and inbound map. There was no difference in slopes between the outbound and inbound maps, for cells with fields included on both maps (t(537)=1.43, p=0.15; outbound=-2.78±5.24, inbound=-3.07±4.89 rad/field)

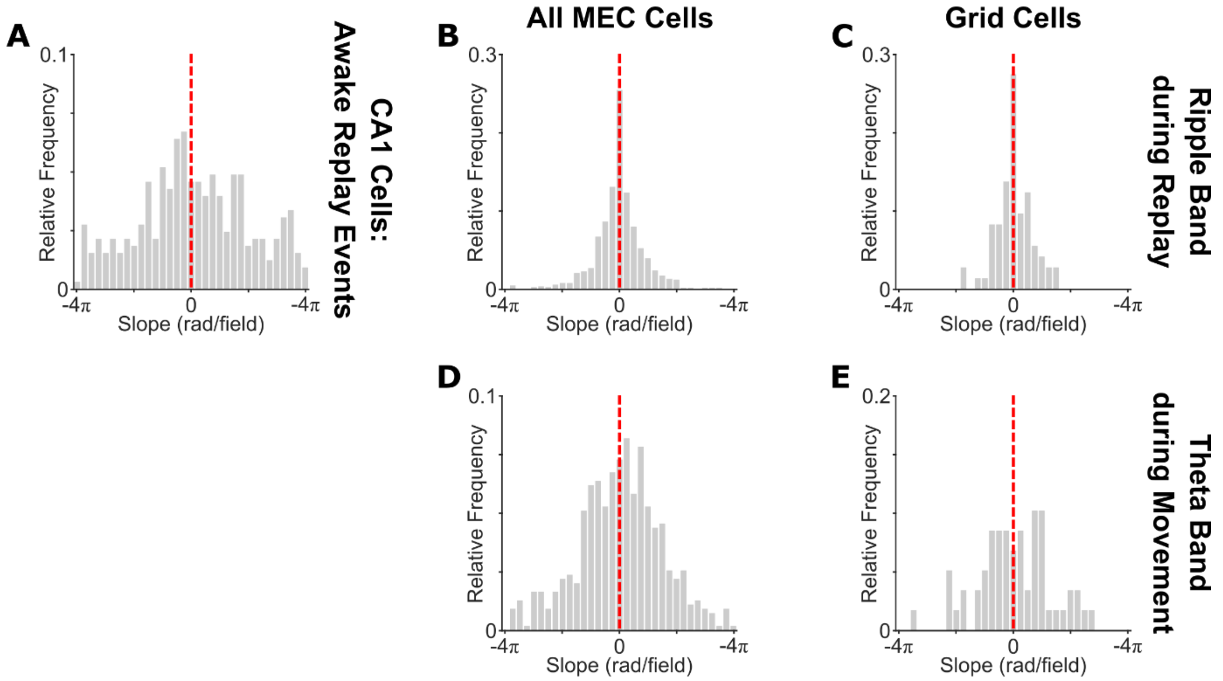

Figure S5: Within-Field Location vs. Firing Phase Relationships during Online Replay Events and in MEC, Related to Figure 4

[A] Within-field location vs. ripple band firing phase slopes across CA1 place cells during online candidate replay events detected from increased MUA. A total of 1177/5590 online replay events corresponded to significant linear trajectories on the track (20±9.16% events per session); and a total of 721 place fields subsequently passed our threshold for inclusion in this analysis (≥5 spikes covering ≥50% of the firing field), with 360/1044 cells (34.5%) having ≥1 field included and 12±12.3 spikes per field (range 5-115). The distribution of slopes across cells (-0.289±6.97 rad/field) does not differ from zero (t(359)=0.067, p=0.95)

[B] Within-field location vs. ripple band firing phase slopes across putative principal cells in MEC during offline candidate replay events detected from increased MUA. A total of 7211/67540 offline replay events corresponded to significant linear trajectories on the track (10.9±3.11% events per session); and a total of 2442 firing fields subsequently passed our threshold for inclusion in this analysis (≥5 spikes covering ≥50% of the firing field), with 572/877 cells (65.2%) having ≥1 field included and 16±37.4 spikes per field (range 5-602). The distribution of slopes across cells (0.299±4.65 rad/field) does not differ from zero (t(550)=0.326, p=0.744)

[C] Within-field location vs. ripple band firing phase slopes across MEC grid cells during offline candidate replay events detected from increased MUA. A total of 59/877 grid cells passed our criteria for inclusion in this analysis. The distribution of slopes across these cells (-0.00628±4.02 rad/field) does not differ from zero (t(58)=0.310, p=0.758)

[D] Within-field location vs. theta firing phase slopes across putative principal cells in MEC during RUN. A total of 945/1033 cells A total of 5498 firing fields passed our threshold for inclusion in this analysis (≥5 spikes covering ≥50% of the firing field), with 946/1033 cells (91.6%) having ≥1 field included and 65±128 spikes per field (range 5-1251). The distribution of slopes across cells (-0.00628±2.56 rad/field) does not differ from zero (t(944)=-0.753, p=0.452)

[E] Within-field location vs. theta firing phase slopes across MEC grid cells during RUN. A total of 73/1033 grid cells passed our criteria for inclusion in this analysis. The distribution of slopes across these cells (0.0792±2.03 rad/field) does not differ from zero (t(72)=0.344, p=0.732). However, 78/536 grid fields (14.6%) showed a significant negative correlation between within-field location and theta phase, which is more than expected by chance (binomial test, p<0.001)
